# The Chloroplast Land Plant Phylogeny: Analyses Employing Better-Fitting Tree- and Site-Heterogeneous Composition Models

**DOI:** 10.3389/fpls.2020.01062

**Published:** 2020-07-10

**Authors:** Filipe Sousa, Peter Civáň, Peter G. Foster, Cymon J. Cox

**Affiliations:** ^1^ Centro de Ciências do Mar, Universidade do Algarve, Faro, Portugal; ^2^ INRA, Université Clermont-Auvergne, Clermont-Ferrand, France; ^3^ Department of Life Sciences, Natural History Museum, London, United Kingdom

**Keywords:** land plants, phylogeny, bryophytes, chloroplast, composition heterogeneity

## Abstract

The colonization of land by descendants of charophyte green algae marked a turning point in Earth history that enabled the development of the diverse terrestrial ecosystems we see today. Early land plants diversified into three gametophyte-dominant lineages, namely the hornworts, liverworts, and mosses, collectively known as bryophytes, and a sporophyte-dominant lineage, the vascular plants, or tracheophytes. In recent decades, the prevailing view of evolutionary relationships among these four lineages has been that the tracheophytes were derived from a bryophyte ancestor. However, recent phylogenetic evidence has suggested that bryophytes are monophyletic, and thus that the first split among land plants gave rise to the lineages that today we recognize as the bryophytes and tracheophytes. We present a phylogenetic analysis of chloroplast protein-coding data that also supports the monophyly of bryophytes. This newly compiled data set consists of 83 chloroplast genes sampled across 30 taxa that include chlorophytes and charophytes, including four members of the Zygnematophyceae, and land plants, that were sampled following a balanced representation of the main bryophyte and tracheophyte lineages. Analyses of non-synonymous site nucleotide data and amino acid translation data result in congruent phylogenetic trees showing the monophyly of bryophytes, with the Zygnematophyceae as the charophyte group most closely related to land plants. Analyses showing that bryophytes and tracheophytes evolved separately from a common terrestrial ancestor have profound implications for the way we understand the evolution of plant life cycles on land and how we interpret the early land plant fossil record.

## Introduction

It is widely accepted that land plants, or embryophytes, descend from an aquatic green algal ancestor (Karol, 2001; McCourt et al., 2004) that colonized land over 450 Mya (Magallón et al., 2013; Morris et al., 2018), however, reconstructing the relationships among the bryophytes (liverworts, hornworts, and mosses) and tracheophytes (lycopods, ferns, and seed plants), and identifying the algal lineage that is most closely related to the embryophytes, has been challenging and controversial (Cox, 2018). These six major land plant lineages, as well as the six major streptophyte algal groups (Klebsormidales, Chlorokybales, Mesostigmales, Coleochaetales, Charales, and Zygnematales) are each typically well-supported clades and considered monophyletic natural groups. Relationships among the streptophyte algae have been determined with increasing congruence and statistical confidence, converging on a phylogeny that places the conjugating algae of the Zygnematophyceae as the sister-group of land plants (Wickett et al., 2014; Puttick et al., 2018). Among the land plants, the monophyly of the tracheophytes is well supported by molecular evidence and has been assumed partly due to their common possession of an elaborate vascular system, although it is now known that the water-conducting cells of bryophytes are homologous to those of tracheophytes and governed by a similar developmental system (Xu et al., 2014). By contrast, a common origin of the three bryophyte groups, independent of the tracheophytes, has not previously been considered likely, with the majority of studies showing that the tracheophytes evolved from the bryophytes after their initial diversification. Indeed, phylogenetic inferences of sequence data from the nuclear, plastid, and mitochondrial genomes have resulted in conflicting yet statistically well-supported topologies of land plant relationships showing that either the liverworts (e.g. Lewis et al., 1997; Gao et al., 2010), the hornworts (e.g. Hedderson et al., 1996; Wickett et al., 2014), or the clade Setaphyta (Puttick et al., 2018), that contains mosses plus liverworts (e.g. Nishiyama and Kato, 1999; Karol et al., 2010), were the first lineage to split from the remaining land plants. However, in recent years, several studies have supported a hypothesis whereby the first divergence of land plants was between bryophytes and tracheophytes, ruling out a direct descendance of the tracheophytes from bryophytes, and having profound implications for how we view the evolution of plants on land. These newer studies have used better-fitting models that more accurately account for heterogeneity in the data, and therefore suggest that previous hypotheses were based on overly simplistic analyses (Cox et al., 2014; Puttick et al., 2018; Sousa et al., 2019).

Incongruence among phylogenetic tree topologies can be attributed to biological processes, such as incomplete lineage sorting (ILS) and hybridization, and methodological issues, such as inappropriate choice of substitution models. In the case of the land plant phylogeny, however, two main evolutionary processes underlie the observed inconsistency of phylogenetic inferences. Firstly, given the large geological timescale over which land plants have evolved, nucleotide data are subject to substitutional “saturation” at synonymous codon sites, that are under low selective pressure since they do not change the amino acid sequence. Over time, multiple substitutions can occur on synonymous sites, to an extent that they no longer carry reliable phylogenetic signal (Jeffroy et al., 2006). In such cases, the exclusion or recoding of synonymous sites is necessary to remove the non-phylogenetic signal (Cox et al., 2014; Sousa et al., 2019). Secondly, sequence data from highly divergent lineages often display compositional heterogeneity, meaning that the long-term probability of change to a particular nucleotide or amino-acid is different among sites or lineages. Consequently, commonly used substitution models, that assume a fixed nucleotide or amino acid composition among all sites and lineages, may lead to erroneous phylogenetic inference if the data are composition heterogeneous (Foster, 2004). Both composition site- and tree-heterogeneity are the result of varying mutational pressures or selection (for example, for high GC content) and may result in a high level of homoplasy. Composition site-heterogeneity can be modeled using mixture models such as the CAT model (Lartillot and Philippe, 2004), whereas composition tree-heterogeneity can be modeled with non-stationary models such as the NDCH model (Foster, 2004; Foster et al., 2009).

In this study, we reassess the support for land plant relationships based on a newly compiled data set of 83 chloroplast protein-coding genes. Chloroplast sequence data typically represents a single linkage group, since chloroplasts are usually inherited uniparentally as a circular non-recombining chromosome, resulting in reduced opportunities for recombination between different chloroplast lineages (Birky, 2001). There are also no documented cases of lateral gene transfer between chloroplast genomes (Bock, 2010). Thus, there is a reasonable expectation that all genes in the chloroplast genome should carry phylogenetic signal supporting the same tree, i.e. the whole chloroplast genome tree is effectively a gene tree which may or may not be congruent with the species tree, and incongruence among trees inferred from individual chloroplast genes is likely the result of systematic error, rather than ILS. The concatenation of chloroplast genes for phylogenetic analyses is therefore justified, and the resulting tree is analogous to a tree reconstructed from a single non-recombining nuclear DNA sequence. However, as in nuclear genomes, chloroplast protein-coding genes are also subject to composition biases due to drift and different mutational pressures, and thus appropriate modeling of composition site- and tree-heterogeneity is warranted for phylogenetic reconstruction from highly-divergent chloroplast sequences.

Our reconstruction of the land plant phylogeny based on codon-degenerated (non-synonymous) nucleotide data and amino acid data, under better-fitting composition tree-heterogeneous (non-stationary) models, result in trees where bryophytes are monophyletic, strengthening the hypothesis presented by Cox et al. (2014). These new analyses, together with published analyses of nuclear protein coding data (Puttick et al., 2018; Sousa et al., 2019) support the hypothesis whereby the first evolutionary split among land plants occurred between the bryophytes and the tracheophytes, and suggests a need for a re-interpretation of the fossil evidence and the nature of the ancestral embryophyte.

## Materials and Methods

The thirty taxa selected for analyses include four chlorophyte algae, nine charophyte algae, of which four are members of the Zygnematophyceae, six bryophytes, sampled evenly among liverworts, mosses, and hornworts, and 11 tracheophytes, including representatives of lycopods, ferns, and seed plants (**Table 1**). Protein-coding genes which were annotated in at least 15 of the sampled taxa were selected for analysis, resulting in a data set of 83 genes (**Supplementary Information Table S1**). Individual nucleotide alignments and the respective amino acid translation were constructed using TranslatorX (Abascal et al., 2010), and poorly aligned regions were identified using GBlocks (vers. 0.91b; Talavera and Castrasana, 2007). Alignments were inspected manually, and regions of low coverage, i.e., at the beginning and ends of sequences, or with ambiguous alignment, were identified and removed by codon triplet position, to maintain a full correspondence between codon triplets of the nucleotide sequences and their amino acid translation. Concatenated data matrices were constructed from the combined protein-coding genes (48861 sites) and their corresponding combined protein translations (16287 sites). The proportion of missing characters among ingroup taxa were very low, with a mean of 4.38% per taxon (median 2.36%), suggesting that the results were unlikely to be biased by ambiguous data (Lemmon et al., 2009). In addition to standard DNA coding, all synonymous substitutions of the protein-coding gene data were eliminated by codon-degenerate recoding with IUPAC ambiguity codes (Cox et al., 2014). Thus, three concatenated data sets were generated: 1) nucleotides, 2) codon-degenerate recoded nucleotides, and 3) the translated amino acid sequences.

**Table 1:** Taxon sampling.

Taxon name	Classification^1^	GenBank Accession	No. of genes^2^	% Missing characters^3^	% G-C
*Chlorella vulgaris*	Chlorophyta, Trebouxiophyceae	NC_001865	65	21.28	38.24
*Chlamydomonas reinhardtii*	Chlorophyta, Chlorophyceae	NC_005353	57	32.39	36.30
*Ostreococcus tauri*	Chlorophyta, prasinophytes	NC_008289	54	35.56	42.02
*Nephroselmis olivacea*	Chlorophyta, prasinophytes	NC_000927	74	4.81	43.13
*Mesostigma viride*	Streptophyta, Mesostigmales	NC_002186	79	3.55	33.57
*Chlorokybus atmophyticus *	Streptophyta, Chlorokybales	NC_008822	81	1.66	37.96
*Klebsormidium flaccidum *	Streptophyta, Klebsormidiales	NC_024167	73	7.23	43.33
*Chara vulgaris*	Streptophyta, Charales	NC_008097	81	1.34	34.63
*Chaetosphaeridium globosum*	Streptophyta, Coleochaetales	NC_004115	83	0.09	33.77
*Staurastrum punctulatum*	Streptophyta, Desmidiales	NC_008116	81	1.22	35.77
*Zygnema circumcarinatum*	Streptophyta, Zygnematales	NC_008117	81	0.90	37.93
*Mesotaenium endlicherianum*	Streptophyta, Zygnematales	NC_024169	81	0.74	44.29
*Roya anglica*	Streptophyta, Zygnematales	NC_024168	81	0.47	36.63
*Pellia endiviifolia*	Streptophyta, Marchantiophyta	NC_019628	82	0.50	38.24
*Ptilidium pulcherrimum*	Streptophyta, Marchantiophyta	NC_015402	77	10.7	35.72
*Physcomitrella patens*	Streptophyta, Bryophyta	NC_005087	80	2.84	33.46
*Syntrichia ruralis*	Streptophyta, Bryophyta	NC_012052	77	9.90	33.21
*Nothoceros aenigmaticus*	Streptophyta, Anthocerotophyta	NC_020259	81	2.80	39.10
*Anthoceros formosae*	Streptophyta, Anthocerotophyta	NC_004543	81	1.92	37.31
*Isoetes flaccida*	Streptophyta, Lycopodiophyta	NC_014675	79	3.83	40.75
*Huperzia lucidula*	Streptophyta, Lycopodiophyta	NC_006861	83	0.03	38.98
*Selaginella moellendorffii*	Streptophyta, Lycopodiophyta	NC_013086	66	10.12	50.77
*Equisetum hyemale*	Streptophyta, Moniliformopses	NC_020146	81	0.52	36.02
*Psilotum nudum*	Streptophyta, Moniliformopses	KC117179	79	7.68	38.57
*Angiopteris evecta*	Streptophyta, Moniliformopses	NC_008829	83	0.01	38.01
*Adiantum capillus-veneris *	Streptophyta, Moniliformopses	NC_004766	79	7.31	43.42
*Pinus thunbergii*	Streptophyta, Spermatophyta	NC_001631	69	20.61	40.65
*Cycas revolute*	Streptophyta, Spermatophyta	JN867588	81	1.08	40.72
*Arabidopsis thaliana*	Streptophyta, Spermatophyta	NC_000932	76	8.70	39.04
*Nymphaea alba*	Streptophyta, Spermatophyta	NC_006050	77	8.20	41.01

Taxon names, classification, NCBI GenBank accession numbers, and numbers of genes present in data set. ^1^Classification follows NCBI GenBank. The given ranks are not equal but the highest available while distinguishing among the six major lineage of land plants and the major groups of algae. ^2^Number of gene present out of a total of 83. ^3^Percentage of missing characters in gene sequence and protein data matrices.

Three tree-independent tests of model process homogeneity were performed using pairwise sequence comparisons in each of the three data sets to assess whether the data were homogeneous with respect to among-lineage composition (i.e. stationarity) and instantaneous substitution rate, and process reversibility. Bowker's Test (Ababneh et al., 2006; Jermiin et al., 2017) is a general test of model process homogeneity between sequences, whereas Stuart's and Ababneh's tests indicate deviation from stationarity and rate homogeneity, respectively (Ababneh et al., 2006; Jermiin et al., 2020; Jermiin and Misof, 2020). All tests were performed using P4 (vers. 0.89 - Foster, 2004).

Optimal sets of partitions among genes (11 partitions) and among codon-positions in genes (21 partitions) were determined using PartitionFinder (Lanfear et al., 2014), using a general time-reversible (GTR) model with a discrete (4 categories) gamma-distribution of rates among sites (Γ_4_), with empirical base frequencies (F_emp_), and with the best partitioning schemes chosen using the Bayesian Information Criterion (BIC). To test whether the optimal gene partitioning scheme estimated by PartitionFinder was dependent on the estimated neighbor-joining starting tree, which by default resulted in a tree in which hornworts were nested in the tracheophytes and is likely incorrect, an alternative optimal gene partitioning scheme, contingent on a fixed tree showing monophyletic bryophytes, was determined and analyzed by ML bootstrap.

Best-fitting substitution models were determined using Modelgenerator (Keane et al., 2006). In addition, the green-plant specific empirical amino-acid substitution model, gcpREV, was used for analyses of amino acid sequence data (Cox and Foster, 2013). Maximum-likelihood (ML) bootstrap analyses were conducted using an MPI-compiled version of RAxML (vers. 7.0.4–7.8.4–8.0.26; Stamatakis, 2006). RAxML analyses consisted of 300 or 400 bootstrap replicates with default settings for parameter estimation accuracy, a discrete gamma-distribution of among-site rate heterogeneity (4 categories; Γ_4_) and estimated composition frequencies (F_est_).

Bayesian Markov Chain Monte-Carlo (MCMC) analyses were performed using P4 with the NDCH and NDCH2 non-stationary composition models (Cox et al., 2008). Homogeneous (stationary) analyses were performed by defining a single composition vector on the NDCH model (CV1). Composition tree-heterogeneous analyses on the protein data were performed using the NDCH2 model which includes a separate composition vector for each node of the tree. Fit of the model composition to the data was determined using posterior predictive simulations of the χ^2^ statistic of composition homogeneity as implemented in P4 (Foster, 2004). Indicators of poor MCMC performance — low acceptance rates, poor mixing between hot and cold chains, excessively long branch lengths (Brown et al., 2010) were noted. MCMC analyses were also performed using Phylobayes MPI (vers. 1.2f — Lartillot and Philippe, 2004) with the CAT infinite profile mixture model (F_CAT_), which specifically handles composition site-heterogeneity. Posterior predictive tests were applied to Phylobayes analyses to assess model-fit.

Stationarity of MCMC chains was assessed by observing the likelihood of samples (and other parameters) over time, and convergence to the correct posterior probability distribution was determined by running multiple MCMC chains in parallel and calculating the average standard deviation of split support (asdoss) between independent chains. Posterior probabilities (PP) < 0.95 and bootstrap values (BS) < 90% were considered low and indicative of weak support of nodes, whereas larger values were considered strong indicators of clade support. Details of individual analyses, the specific models used, and the diagnostic statistics are included in the legends of the figures in the Supporting Information. The combined nucleotide, codon-degenerate and protein matrices, all in nexus format and with characters sets, were deposited on Zenodo (DOI: 10.5281/zenodo.3886964).

## Results

### Matched-Pairs Tests of Process Homogeneity

**Figure 1:**
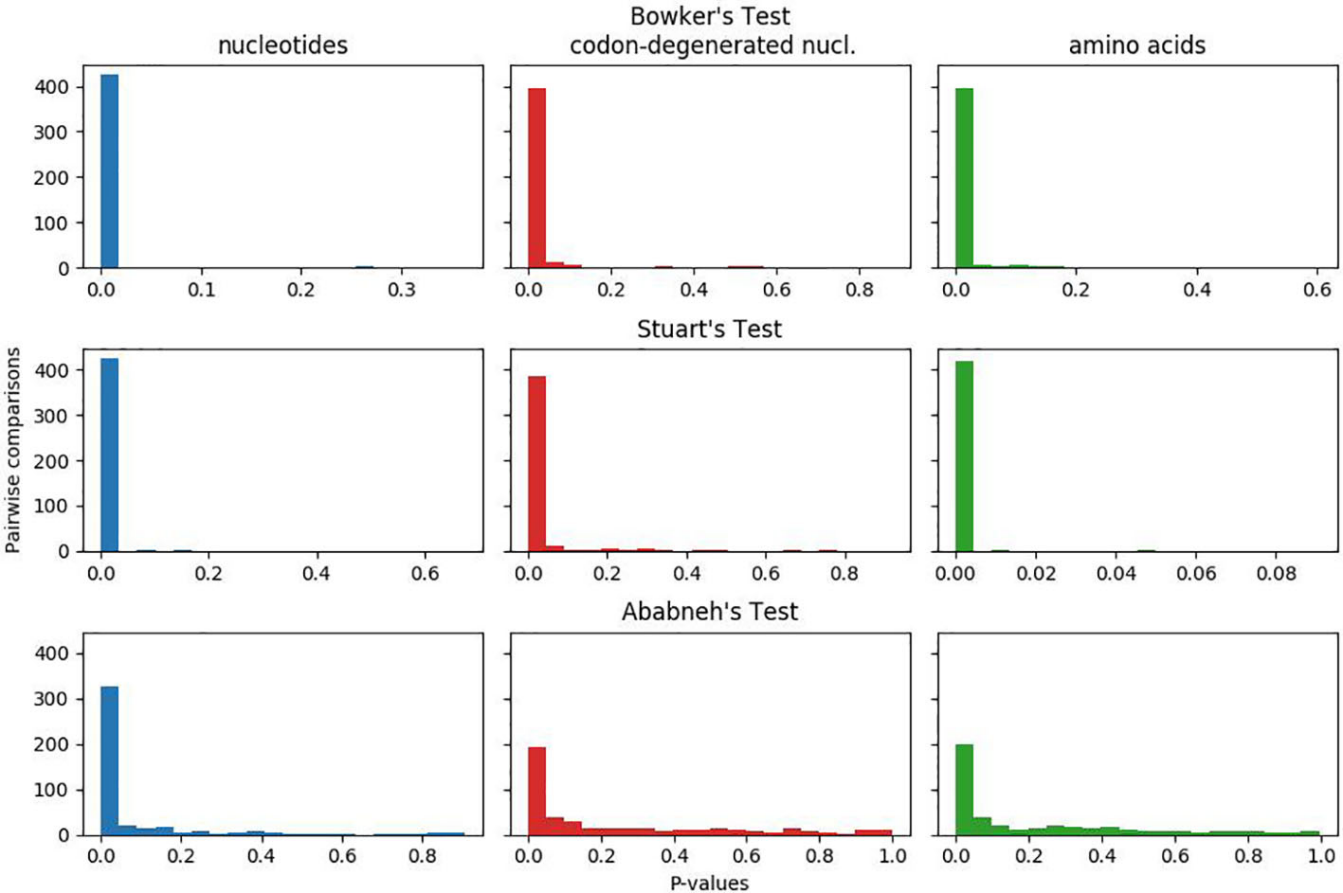
Plots of p-values for Bowker's, Stuart's, and Ababneh's matched-pairs tests of model homogeneity for each of the three data sets. Numbers of rejected (p-values < 0.05) tests: Bowker's test nucleotides 427 (98%), codon-degenerated nucl. 398 (91%), amino acids 401 (92%); Stuart's test nucleotides 424 (97%), codon-degenerated nucl. 387 (89%), amino acids 401 (98%); Ababneh's test nucleotides 331 (76%), codon-degenerated nucl. 193 (44%), amino acids 126 (29%).

In **Figure 1**, the plotted p-values for the matched-pairs tests of homogeneity for each of the nucleotide, codon-degenerated, and amino acid data sets are shown. All three data sets fail all three tests, although the assumption of model homogeneity is violated more severely in the nucleotide data sets than in the amino acids data. These tests indicate that the data are neither stationary with respect to composition (composition varies across lineages) or homogeneous with respect to instantaneous rates (rates vary across lineages).

### Nucleotide Data

**Figure 2:**
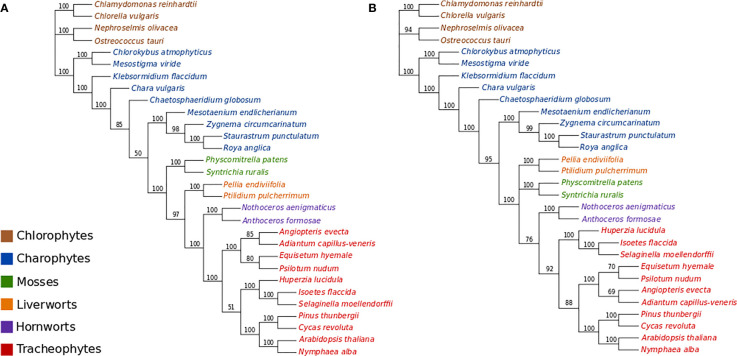
**(A)** ML bootstrap analysis (400 replicates) of the nucleotide data (GTR+Γ_4_). Optimal ML tree score: -ln L= 757308.3933. Length of optimal tree = 10.0 substitutions/site. **(B)** ML bootstrap analyses (400 replicates) of the codon-degenerate recoded nucleotide data (GTR+Γ_4_). Optimal ML tree score: -ln L= 304862.2078. Optimal tree length = 2.7 substitutions/site.

All ML bootstrap analyses of the protein-coding nucleotide data (GTR+Γ_4_ +F_est_) strongly support the placement of the moss lineage as sister-group to all other plants (BS>90%), with the hornworts fully supported as the sister-group to the tracheophytes (**Figure 2A**; **Figures S1–S3**). ML bootstrap analyses with optimal numbers of gene partitions (11 partitions with separate models; **Figure S1**) did not result in topological differences compared to the non-partitioned ML bootstrap analysis (**Figure 2A**), and the use of an alternative starting tree for estimating the optimal gene partitioning scheme resulted in a slightly altered partitioning scheme but ultimately had no substantial effect on the statistical support regarding the placement of bryophyte lineages (**Figure S2**). The ML bootstrap analyses with optimal numbers of codon-position partitions (21 partitions; **Figure S3**) was also congruent with other analyses regarding the placement of bryophytes, but resulted in a different arrangement among tracheophyte lineages. Whereas the non-partitioned and gene-partitioned analyses placed ferns as sister-group to other tracheophytes in the codon-position and partitioned analyses, the lycopods appear as sister-group to other tracheophytes in the codon-partitioned analyses (**Figure S3**).

Bayesian MCMC analyses of the nucleotide data using a tree-homogeneous composition model shows full support (PP = 1.0) for the placement of mosses as sister-group to other land plants and hornworts as the sister-group to tracheophytes (**Figure S4**). Similarly, tree-heterogeneous composition model analyses (F_NDCH2_) resulted in a similar topology and support values, but with the lycopods as the sister-group to the remaining tracheophytes (**Figure S5**). Posterior predictive simulations of the χ^2^ statistic of composition homogeneity showed a poor fit (p = 0.0) of both the tree-homogeneous composition model (F_CV1_) and the tree-heterogeneous composition (F_NDCH2_) model, but the latter was a much improved fit by 2 orders of magnitude (see legends of **Figures S4** and **S5** for details). Site-heterogeneous composition model analyses using the Phylobayes (GTR+Γ_4_+F_CAT_) also placed the mosses as the sister-group to the other land plants, but with low branch support (PP = 0.84), whereas hornworts remained strongly supported as the sister-group to tracheophytes (PP = 1.0; **Figure S6**).

The ML bootstrap analyses of the codon-degenerate recoded nucleotide data set (GTR+Γ_4_+F_est_) did not resolve the position of either mosses or liverworts, and resolved hornworts as sister-group to tracheophytes with low branch support (BS = 76%; **Figure 2B**).

### Amino Acid Data

ML bootstrap analyses of the concatenated amino acid data (gcpREV+Γ_4_+F_mod_) resolve bryophytes as monophyletic (BS = 77%) but fail to recover the monophyly of tracheophytes, showing ferns as the sister-group to the remaining embryophytes but with very low statistic support (BS = 56%; **Figure S7**). When the data were divided into 17 partitions, ML bootstrap support for the monophyly of the bryophytes increased to 81%, and the ferns were supported as the sister-group to all other land plants by 66% (**Figure S8**).

**Figure 3:**
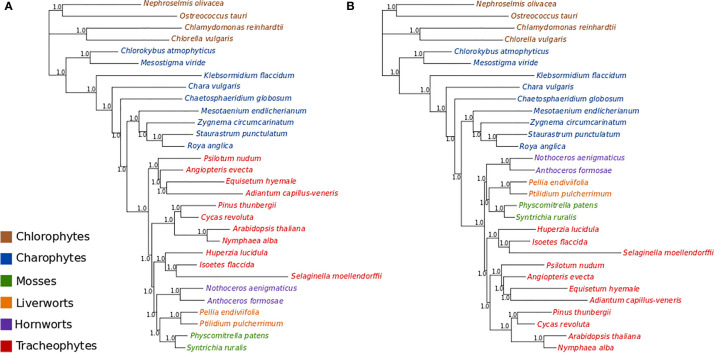
Bayesian MCMC tree-heterogeneous composition analyses of the amino acid data (gcpREV+Γ_4_+F_NDCH2_) **(A)** Run1: Marginal likelihood: L_h_= 302615.5702. Posterior predictive simulations of χ^2^ statistic of composition homogeneity: original statistic = 3021.4465, sample distribution = 2040.7165 to 3069.3241, p-value= 0.0005. 4,000,000 generations, 40,000 samples, 10,000 discarded as burnin. Mean tree length = 5.6538 substitutions/site. **(B)** Run2: Marginal likelihood: L_h_= 302634.1013. Posterior predictive simulations of χ^2^ statistic of composition homogeneity: original statistic= 3021.4465, sample distribution = 2047.7021 to 3081.9710, p-value = 0.0002. 4,000,000 generations, 40,000 samples, 10,000 discarded as burnin. Mean tree length = 5.6872 substitutions/site.

MCMC analyses of amino acid data under a tree-homogeneous composition model (gcpREV+Γ_4_ +F_est_; **Figure S9**) and under the tree-heterogeneous composition model (gcpREV+Γ_4_ +F_NDCH2_; **Figures 3A, B**; **Figures S10** and **11**) both show bryophytes as monophyletic with maximum support (PP = 1.0) in all replicates of the analyses. However, the four independent runs of non-stationary composition (F_NDCH2_) analyses failed to converge on the same topology with respect to the relationships among the tracheophyte lineages. In two runs (runs 1 and 3, **Figures 3A** and **S10**, respectively), including the run with the best marginal-likelihood score (run 1), tracheophytes were resolved as paraphyletic, with ferns placed as sister-group to the remaining embryophytes and lycopods as sister-group to the bryophyte clade. The two other runs (runs 2 and 4, **Figures 3B** and **S11**, respectively) recovered tracheophytes as monophyletic, with lycopods as the sister-group to the clade containing ferns and seed plants. All nodes on the trees obtained from every run received maximum support (PP = 1.0). Neither the tree-homogeneous or the tree-heterogeneous (F_NDCH2_) composition model fit the data, according to posterior predictive simulations of χ^2^, but the NDCH2 model was a much better approximation than the homogeneous model as the test statistic fell within the sample distribution of the runs, albeit outside the 95% confidence interval (see legends of **Figures S10** and **S11** for details).

**Figure 4:**
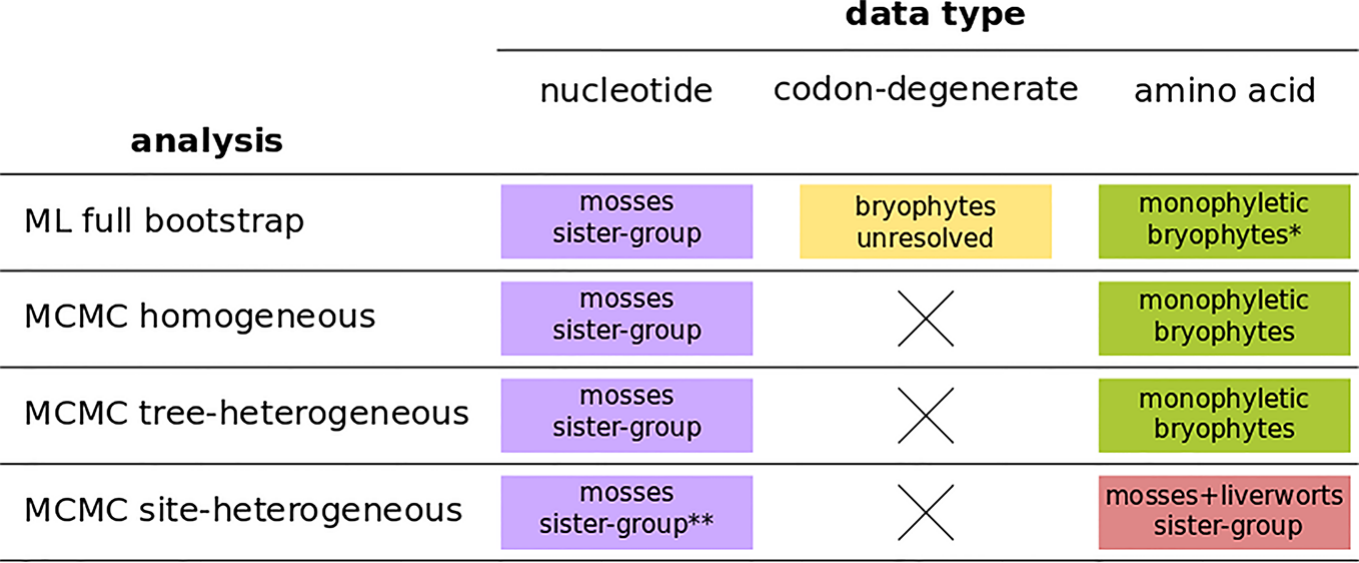
A summary of bryophyte relationships obtained from nucleotide, codon-degenerate, and amino acid translation data using Maximum-likelihood bootstrap analyses (ML) and Bayesian (MCMC) homogeneous, tree-heterogeneous, and site-heterogeneous analyses. Tree nodes were considered supported if the bootstrap value was equal or higher than 80% or if the posterior probability was equal or higher than 95%. (*) part of the analyses without node support; (**) no node support.

The four independent MCMC analyses of the amino acid data with the site-heterogeneous composition model (GTR+G+F_CAT_) resulted in trees showing the clade Setaphyta as the sister-group to the remaining land plants (PP = 0.96–0.98) and hornworts as the sister-group of tracheophytes (**Figures S12–S15**). Posterior predictive tests of the four runs showed that all but one run passed the site diversity test that estimates the fit of the model to describe the mean number of distinct amino acids per site. However, the null hypothesis was rejected (i.e. the model does not fit the data adequately) by posterior predictive simulations in other tests: a) the empirical convergence probability test which estimates the long-term probability of two sites converging on the same character state in two random taxa; b) across-site compositional heterogeneity test; c) across-taxa maximum heterogeneity test; and d) across-taxa mean squared heterogeneity test. Additional MCMC runs on amino acid data with constant sites removed, previously thought to influence tree topology in these analyses, did not show differences in topology or branch support (not shown). A summary of bryophyte relationships obtained from each data type, and each type of analysis is presented in **Figure 4**.

The conjugating algae group Zygnematophyceae was resolved as sister-group to land plants in all but one analysis where the sister-group to land plants was not resolved (**Figure S2**). When resolved, this relationship received high to maximum branch support (BS between 80% and 100%, PP > 0.95) except for analyses of nucleotide data with composition tree-homogeneous models (ML, **Figures S1** and **S3**; Bayesian MCMC, **Figure S4**) and with a site-heterogeneous composition (F_CAT_) model (**Figure S6**).

## Discussion

### Taxon Sampling and Model Fit

The chloroplast phylogeny of land plants and streptophytes has been inferred many times previously using different data and analytical approaches, but these studies have often resulted in conflicting phylogenetic patterns (e.g. Nishiyama et al., 2004; Chang and Graham, 2011; Ruhfel et al., 2014). However, almost all studies neglect to test the adequacy of the models used to reconstruct the phylogeny, while others fail even to report the model used (e.g. Gitzendanner et al., 2018). Here we highlight and distinguish between model-fit and model adequacy: while nearly all studies test model fit to choose a “best” substitution model from among a selection, model adequacy is concerned with how well the model fits the data in absolute terms — a best-fitting model may still be a very poor fit to the data. The three matched-pairs tests we conducted (**Figure 1**) show that all three data sets are neither composition nor rate homogeneous through time. Therefore tree-homogeneous models are likely to be a very poor fit to the data, and yet such models have been, and continue to be, widely used as the only means of reconstructing the phylogeny of land plants. In this paper we employed tree-heterogeneous composition (NDCH2) and site-heterogeneous composition (CAT) model analyses, but to date no single analyses have been conducted that account for both process, and no analyses that account for among-lineage rate variation have been conducted. We identify here that all three processes are likely important for the accurate reconstruction of the land plant phylogeny.

Two studies are notable for having used whole chloroplast genome data together with substitution models that account for composition heterogeneity across sites (Cox et al., 2014) and across taxa (Lemieux et al., 2016). The work of Cox et al. (2014) used a tree-heterogeneous composition model in both nucleotide and amino acid data, showing that amino-acid data support the monophyly of bryophytes, and that when synonymous substitutions are eliminated, support for the non-monophyly of bryophytes is lost in nucleotide data. By contrast, in the work by Lemieux et al. (2016), the analyses of amino acid data using a site-heterogeneous composition model instead showed maximum support for the placement of Setaphyta alone as sister-group to the remaining land plants with the hornworts the sole sister-group to the tracheophytes. In both data sets, sampling of bryophyte lineages was limited, with only one representative of hornworts and one of liverworts. In addition, the data set of Cox et al. (2014) had a very imbalanced proportion of bryophytes and tracheophytes (4:33), which may affect phylogenetic reconstruction, and the data set of Lemieux et al. (2016) lacked any representative of the ferns. The present data set aimed at correcting this sampling bias, and included two representatives of each bryophyte lineage, as well as a balanced representation of each tracheophyte lineage, including ferns. The two taxa for each of the bryophyte lineages were chosen (where possible) to span the likely ancestral node of the lineage with the intention of more accurately reconstructing ancestral states and reducing the length of the subtending branches. By doing this, the genetic distances between lineages were minimized and the likelihood of long-branch attraction reduced. There was a conscious decision to limit the numbers of taxa sampled while sampling as much data as was computationally tractable. Even so, the most complex Bayesian MCMC tree-heterogeneous composition (NDCH2) analyses took > 6 months single CPU computational time to complete per analytical run.

Recent maximum-likelihood analyses of protein-translated plastid transcriptome data spanning the entire green plant kingdom resulted in trees showing the monophyly of bryophytes (Gitzendanner et al., 2018; Leebens-Mack et al., 2019); a result similar to that presented here. However, these studies did not evaluate whether the time-homogeneous models they used in their studies were an adequate fit to their data. This is especially important as Cox et al. (2014) (as again in this study) have shown that land plant plastid data are time-heterogeneous, and therefore the results of these studies are difficult to interpret as they may by compromised by their use of poor-fitting time-homogeneous models.

### Conflict Between Nucleotide and Amino Acid Chloroplast Data Is Reduced When Synonymous Substitutions Are Excluded

One common technique used to reduce the probability of systematic errors in phylogenetic reconstruction is to remove data that cannot be adequately modeled, thus increasing the fit of the model and the likely accuracy of the reconstructed trees (e.g. Goremykin et al., 2003). With time, a proportion of site characters uniting a lineage (synapomorphies) are inevitably erased by multiple substitutions and are said to be "saturated” when all phylogenetic signal is lost. Saturation in a protein-coding gene sequence can be reduced by eliminating substitutions which represent synonymous amino-acid replacements. These substitutions occur more rapidly than non-synonymous substitutions as they are not constrained by protein structure and function and therefore are less likely to reflect accurate phylogenetic signal. By removing synonymous substitutions from the nucleotide data, the tree length was reduced from a very high estimated substitution rate of 9.9 substitutions per site to only 2.7 substitutions per site (**Figures 2A, B**). However, while using degenerate ambiguity recoding to eliminate synonymous substitutions can reduce the amount of non-historical signal present in the data, it does not eliminate it as composition biases can still be caused by different selective pressures for amino acids at protein sites and due to mutational biases. In our analyses, we show that excluding synonymous substitutions eliminates signal in the nucleotide data that supports mosses as sister-group to embryophytes and decreases support for the grouping of hornworts and tracheophytes. Consequently, we think it likely that support for the non-monophyly of the bryophytes in nucleotide sequences is due to non-historical signal (substitutional saturation) present in synonymous sites.

### Composition Tree-Heterogeneous Analyses of Chloroplast Amino-Acid Data Support the Monophyly of Bryophytes

ML and Bayesian analyses of chloroplast protein data tend to support the monophyly of the bryophytes (**Figures S9–S15**), however this support is sometimes coincident with the non-monophyly of the tracheophytes. The non-monophyly of the tracheophytes in Bayesian homogeneous and tree-heterogeneous composition analyses, and indeed the implication that tracheophytes are ancestral to bryophytes, is a result that has not been reported before. The topologies where tracheophytes are paraphyletic with the ferns as the earliest-diverging lineage of all land plants, or the ferns are the earliest-diverging lineage of the tracheophytes alone, are almost certainly inaccurate. This is because both the ferns and seed plants share a unique 30-kb inversion in the large, single copy region of the chloroplast genome that is very likely a unique character uniting ferns and seed plants to the exclusion of other taxa as it is thought unlikely that such a structural rearrangement could be reversed (Raubeson and Jansen, 1992). The non-canonical early-branching of the fern lineage suggests that the ferns are being drawn toward the base of the land plants, possibly as an artifact caused by among-site compositions heterogeneity as Phylobayes CAT analyses strongly support the monophyly of the tracheophytes. Unfortunately, the better-fitting Bayesian tree-heterogeneous composition analyses were inconclusive with identical tree topologies having varying marginal likelihood scores: of 4 replicate runs the tracheophyte-paraphyletic topology scored both the best and 3rd best marginal likelihoods, while the tracheophyte-monophyletic topology scored both the 2^nd^ and 4^th^ best marginal likelihoods. This suggests that the composition values sampled at nodes are the critical factor, and not the topology, and that the mixing of the MCMC chains was not efficient enough to allow independent runs to converge to a single best solution.

By contrast, the analyses using composition site-heterogeneous models (Phylobayes CAT) support the Setaphyta as the earliest branching lineage with the hornworts as the sister-group to the tracheophytes. Here the monophyly of the tracheophytes is maximally supported, suggesting that perhaps the modeling of among-site composition heterogeneity is critical to resolving the tracheophytes with amino-acid data. However, posterior predictive tests of the CAT model showed that it failed to describe data heterogeneity across both sites and lineages, with a particularly strong rejection (high Z-scores) of the null hypothesis for the among-lineage composition heterogeneity test. Nevertheless, these analyses suggest the paraphyly of bryophytes, with hornworts the sister-group to tracheophytes, may be the result of among-lineage composition biases as the NDCH2 analyses show strong support for the monophyly of bryophytes. Indeed it may be that among-lineage composition heterogeneity is critical to resolving the monophyly of the bryophytes while at the same time among-site heterogeneity is critical to resolving the monophyly of tracheophytes in these data. Unfortunately, while models combining both these facets of the substitution process are available (e.g. NHPhylobayes, Blanquart and Lartillot, 2006 they are currently computationally intractable with a data set of this size.

### The First Land Plants

While the analyses presented here for chloroplast data are inconclusive as to the relationships among the major lineages of plants, they do support bryophytes as a monophyletic group under tree-heterogeneous models. This observation is in accord with some recent analyses of nuclear data (Puttick et al., 2018; Sousa et al., 2019), but not the mitochondrial data (Liu et al., 2014; Sousa et al., 2020). The conclusion that bryophytes are monophyletic, and therefore that tracheophytes are not derived from a bryophyte ancestor, changes our perspective on trait evolution at the stem of the land plants. Indeed, a phylogeny wherein tracheophytes and bryophytes split from a common terrestrial ancestor implies that the alternation of generations in early land plants was not necessarily identical to that of extant bryophytes (Kenrick, 2017), which has an unbranched sporophyte that is fully dependent on the gametophyte. Instead, even if sporophytes were nutritionally dependent on gametophytes at early stages of development, it is possible that the first land plants had a branched sporophyte, and perhaps even near-isomorphic free-living alternate generations, as in the fossil plants *Horneophyton* and *Aglaophyton*, from the Rhynie chert flora. These are considered early polysporangiophytes (Kenrick and Crane, 1997) but, if tracheophytes are not directly derived from bryophytes, they could perhaps have retained traits from an ancestor that pre-dates the bryophyte-tracheophyte split. Thus, a scenario where both gametophytes and sporophytes possessed the necessary machinery for free-living, and became reduced in the tracheophyte and bryophyte lineages, respectively, is as possible as one where tracheophytes evolved from a simple, heterotrophic sporophyte. The evolution of stomata in land plants, given a monophyletic-bryophytes phylogeny, is not so clear, but they are not a shared-derived character (synapomorphy) uniting the hornworts, mosses, and tracheophytes (Mishler and Churchill, 1984; Ruszala et al., 2011). Only if it is assumed that the probability of loss of stomata was greater than the probability of gain (a not unreasonable assumption, see Harris et al., 2020) then the evolution of stomata would be a synapomorphy uniting all the land plants, with losses in the liverworts and several early-branching moss lineages. Else, if stomata are not homologous among land plants, then they would have been gained independently in the hornworts, mosses, and tracheophytes.

As a corollary to bryophytes forming a monophyletic group, we suggest that a formal classification of the clade containing all three bryophyte lineages as Division (Phylum) Bryophyta Schimp., comprising the three Classes Anthocerotopsida (hornworts), Marchantiopsida (liverworts), and Bryopsida (mosses), will likely have a favorable impact on botanical and evolutionary teaching, as the morphological, reproductive, and ecological traits shared among these three lineages inevitably lead to an intuitive recognition of bryophytes as a natural group.

## Data Availability Statement

The accession numbers for the genomes analyzed in this article can be found in Table 1. Alignment files are available on Zenodo (DOI: 10.5281/zenodo.3886964).

## Author Contributions

CC and PF conceived the study. CC and FS performed analyses. CC, FS, PC, and PF wrote the paper.

## Funding

This work was supported by FCT (Portuguese Foundation for Science and Technology) through project grant PTDC/BIA-EVF/1499/2014 to CC and national funds through project UIDB/04326/2020, and from the operational programs CRESC Algarve 2020 and COMPETE 2020 through projects EMBRC.PT ALG-01-0145-FEDER-022121 and BIODATA.PT ALG-01-0145-FEDER-022231.

## Conflict of Interest

The authors declare that the research was conducted in the absence of any commercial or financial relationships that could be construed as a potential conflict of interest.
