# Supplementary material for: The Chloroplast Land Plant Phylogeny: Analyses Employing Better-Fitting Tree- and Site-Heterogeneous Composition Models
Source: Front Plant Sci. 2020 Jul 10;11:1062. doi: 10.3389/fpls.2020.01062 (PMC7373204; doi:10.3389/fpls.2020.01062)
Supplement: Supplementary file 1 [file DataSheet_1.pdf]

1 Supplemental Information

1.1 Notes

Base compositions of models were estimated from the data, except where indicated they were derived from the model (F_{mod}).

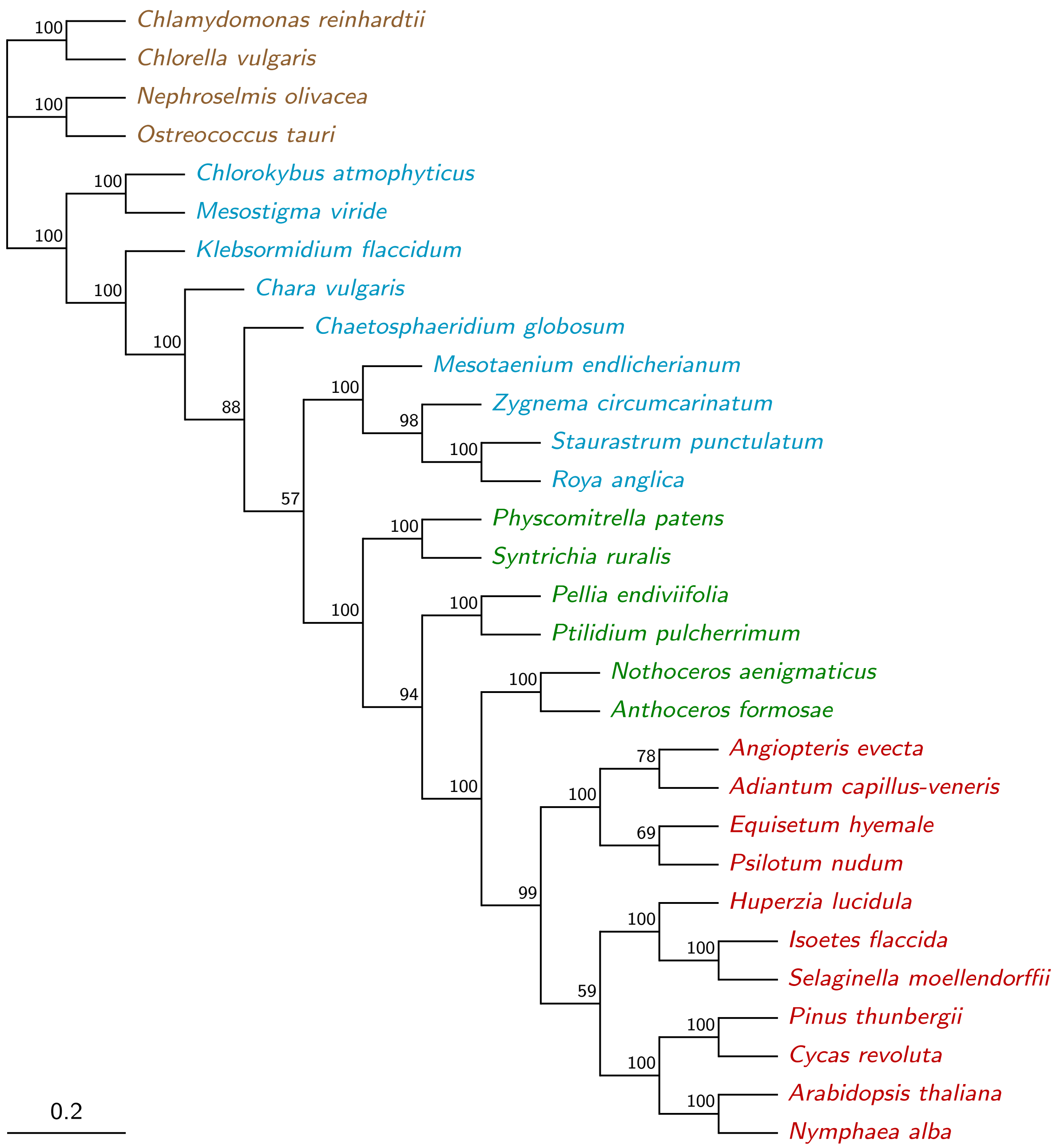

Figure S1 **Protein-coding gene data ML bootstrap: GTR+Γ_4 300 replicates** 11 gene partitions as determined by PartitionFinder: (accd, chlb, infa, peta, psbn, rpl14, ycf3) (atpa, atpb, chll, chln, ndhh, ndhi, ndhk, rps12, rps19) (atpe, rpl16, rpl2, rpl20, rpl21, rpl22, rpl32, rpl33, rpl36, rps14, rps15, rps16, rps3, rps7, rps8) (atpf, matk, rpl23, rpoa) (atph, psac, psbb, psbc, psbd, rbcl) (atpi, ndha, ndhj, petg, psaj, psbh, psbi, psbm, psbz) (ccsa, ndhb, ndhc, ndhd, ndhe, ndhf, ndhg, petl, petn, psai, psam, psbj, ycf12) (cema, clpp, ycf4) (petb, petd, psaa, psab, psbe, psbf, psbk, psbl, psbt) (psba) (rpob, rpoc1, rpoc2, rps11, rps18, rps2, rps4). Optimal ML score: $-lnL$ = 753337.5906. Length of optimal tree = 10.8766

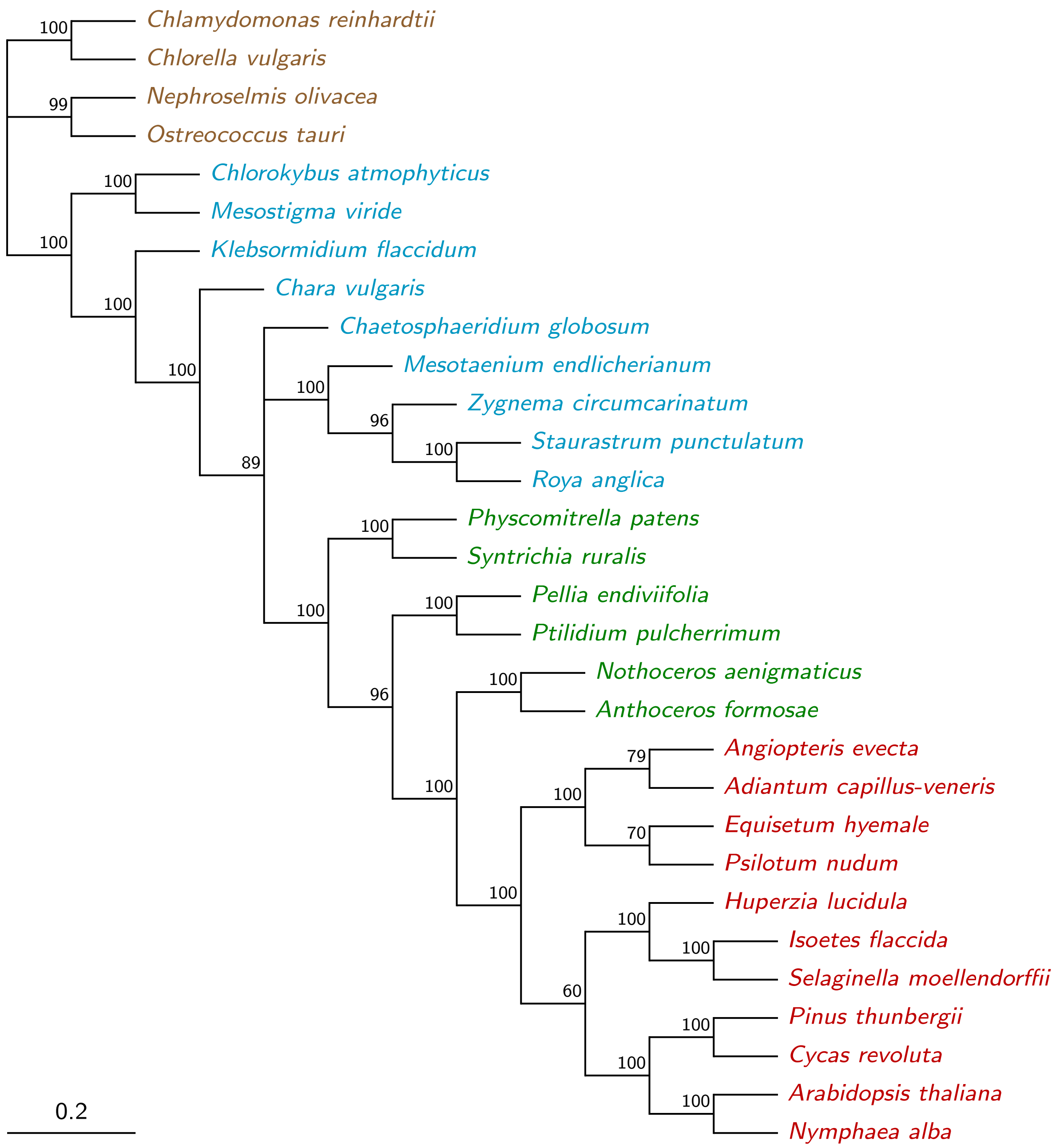

Figure S2 Protein-coding gene data ML bootstrap: GTR+Γ_4 300 replicates 11 gene partitions as determined by PartitionFinder but using a monophyletic bryophytes starting-tree: (accd, chlb, infa, peta, psbn, rpl14, rpl33, rpl36, rps19, ycf3) (atpa, atpb, chll, chln, ndhh, ndhi, ndhk) (atpe, rpl21, rpl22, rpob, rpoc1, rpoc2, rps11, rps14, rps15, rps18, rps2, rps3, rps4, rps8) (atpf, matk, rpl23, rpoa) (atph, psac, psbc, psbd, psbi, rbcl) (atpi, ndha, ndhj, psbh) (ccsa, ndhb, ndhc, ndhd, ndhe, ndhf, ndhg, petl, petn, psai, psam, psbj, ycf12) (cema, clpp, ycf4) (petb, petd, petg, psaa, psab, psaj, psbb, psbe, psbf, psbk, psbl, psbm, psbt, psbz) (psba) (rpl16, rpl2, rpl20, rpl32, rps12, rps16, rps7). Optimal ML score: $-lnL$ = 753338.8832. Length of optimal tree = 10.8490

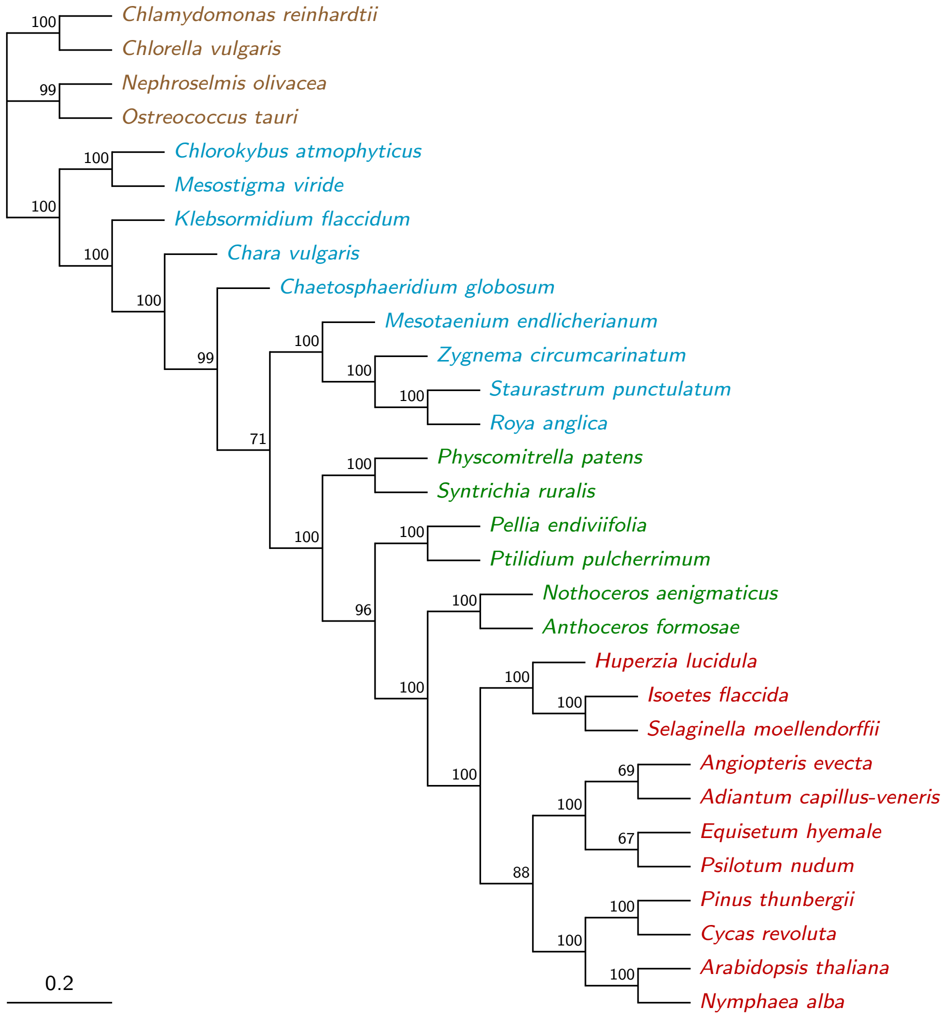

Figure S3 **Protein-coding gene data ML bootstrap: GTR+Γ_4 300 replicates** 21 gene partitions as determined by PartitionFinder: (accd_1pos, chlb_1pos, chln_1pos, ndhh_1pos, ndhi_1pos, ndhj_1pos, peta_1pos, psbj_1pos, psbn_1pos, rpl14_1pos, rps12_1pos, rps19_1pos) (accd_2pos, atpi_2pos, chlb_2pos, chln_2pos, infa_2pos, ndhh_2pos, ndhi_2pos, ndhj_2pos, peta_2pos, psbl_2pos, rpl14_2pos, rpl16_2pos, rpl36_2pos, rps11_2pos, rps18_2pos, rps19_2pos, ycf3_2pos) (accd_3pos, atpb_3pos, atpi_3pos, chlb_3pos, chll_3pos, chln_3pos, ndhh_3pos, ndhi_3pos, ndhj_3pos, ndhk_3pos, petb_3pos, petd_3pos, psac_3pos, psbb_3pos, psbh_3pos, psbi_3pos, rps19_3pos, ycf3_3pos) (atpa_1pos, atpb_1pos, psab_1pos, psac_1pos, psbb_1pos, psbf_1pos, psbk_1pos, rbcl_1pos) (atpa_2pos, atpb_2pos, psab_2pos, psbb_2pos, psbe_2pos, rps12_2pos) (atpa_3pos, cema_3pos, clpp_3pos, infa_3pos, ndha_3pos, peta_3pos, petg_3pos, psaj_3pos, rpl14_3pos, rpl16_3pos, rpl32_3pos, rpl33_3pos, rpl36_3pos, rps12_3pos, rps14_3pos, rps15_3pos, rps16_3pos, rps18_3pos, rps2_3pos, rps3_3pos, rps4_3pos, rps8_3pos, ycf12_3pos) (atpe_1pos, atpf_1pos, clpp_1pos, infa_1pos, rpl16_1pos, rpl20_1pos, rpl21_1pos, rpl22_1pos, rpl23_1pos, rpl2_1pos, rpl36_1pos, rpob_1pos, rpoc1_1pos, rpoc2_1pos, rps11_1pos, rps14_1pos, rps16_1pos, rps18_1pos, rps2_1pos, rps3_1pos, rps4_1pos, rps7_1pos, rps8_1pos, ycf3_1pos) (atpe_2pos, atpf_2pos, clpp_2pos, rpl20_2pos, rpl21_2pos, rpl22_2pos, rpl23_2pos, rpl2_2pos, rpl32_2pos, rpl33_1pos, rpl33_2pos, rpoa_2pos, rpob_2pos, rpoc1_2pos, rpoc2_2pos, rps14_2pos, rps16_2pos, rps2_2pos, rps3_2pos, rps4_2pos, rps7_2pos, rps8_2pos) (atpe_3pos, atpf_3pos, matk_3pos, ndhe_3pos, rpl20_3pos, rpl21_3pos, rpl22_3pos, rpl23_3pos, rpl2_3pos, rpoa_3pos, rpob_3pos, rpoc1_3pos, rpoc2_3pos, rps11_3pos, rps7_3pos) (atph_1pos, chll_1pos, ndhk_1pos, petb_1pos, petd_1pos, petg_1pos, petn_1pos, psaa_1pos, psba_1pos, psbc_1pos, psbd_1pos, psbe_1pos, psbi_1pos, psbl_1pos, psbm_1pos, psbt_1pos) (atph_2pos, chll_2pos, ndhk_2pos, petb_2pos, psaa_2pos, psac_2pos, psba_2pos, psbc_2pos, psbd_2pos, psbi_2pos, rbcl_2pos) (atph_3pos, petl_1pos, petn_3pos, psaa_3pos, psab_3pos, psbc_3pos, psbd_3pos, psbe_3pos, psbf_3pos, psbk_3pos, psbl_3pos, psbt_3pos, rbcl_3pos) (atpi_1pos, ndha_1pos, ndhg_1pos, psai_1pos, psaj_1pos, psbh_1pos, psbz_1pos, ycf12_1pos) (ccsa_1pos, matk_2pos, ndhb_1pos, ndhc_1pos, ndhd_1pos, ndhe_1pos, ndhf_1pos, psam_1pos, rps15_2pos) (ccsa_2pos, ndhb_2pos, ndhd_2pos, ndhe_2pos, ndhf_2pos, ndhg_2pos, petl_2pos, psai_2pos, psam_2pos) (ccsa_3pos, ndhc_3pos, ndhd_3pos, ndhf_3pos) (cema_1pos, matk_1pos, rpl32_1pos, rpoa_1pos, rps15_1pos, ycf4_1pos) (cema_2pos, ycf4_2pos) (ndha_2pos, ndhc_2pos, petd_2pos, petg_2pos, petn_2pos, psaj_2pos, psbf_2pos, psbh_2pos, psbj_2pos, psbk_2pos, psbm_2pos, psbn_2pos, psbt_2pos, psbz_2pos, ycf12_2pos) (ndhb_3pos, ndhg_3pos, petl_3pos, psai_3pos, psam_3pos, psbj_3pos, psbm_3pos, psbn_3pos, psbz_3pos, ycf4_3pos) (psba_3pos). Optimal ML score: $-lnL$ = 728373.7210. Length of optimal tree = 22.0826

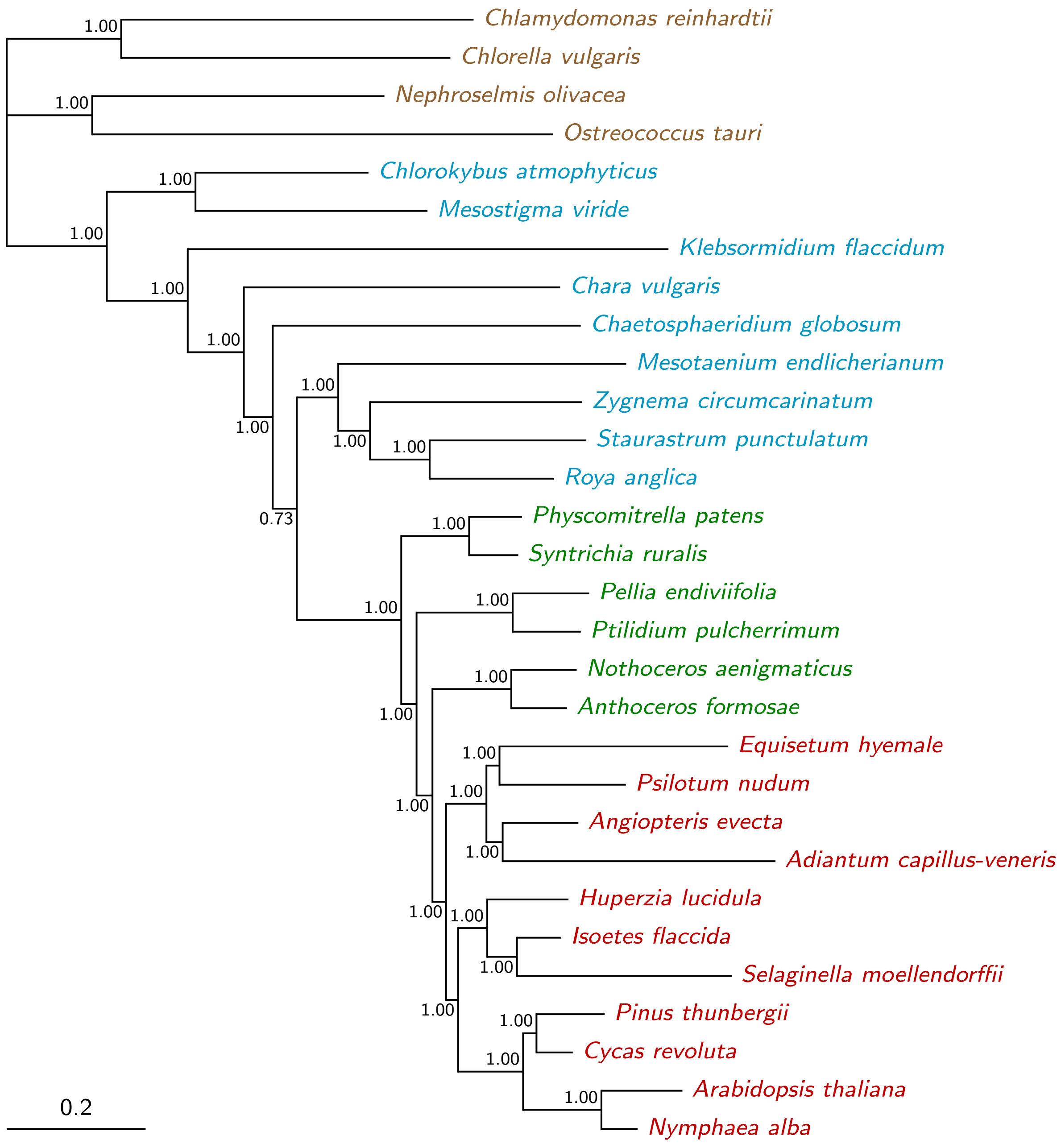

Figure S4 **Protein-coding gene data P4 MCMC homogeneous: GTR+I+Γ_4** Marginal likelihood: $-\overline{L}_h$ = 756517.4688. Posterior predicitive simulations of X^2: original statistic = 9116.6857, sample distribution= 15.9299 to 63.1001, p = 0.0000. 2,000,000 generations, 10,000 samples, 5,000 discarded as burnin. Mean tree length = 9.7321

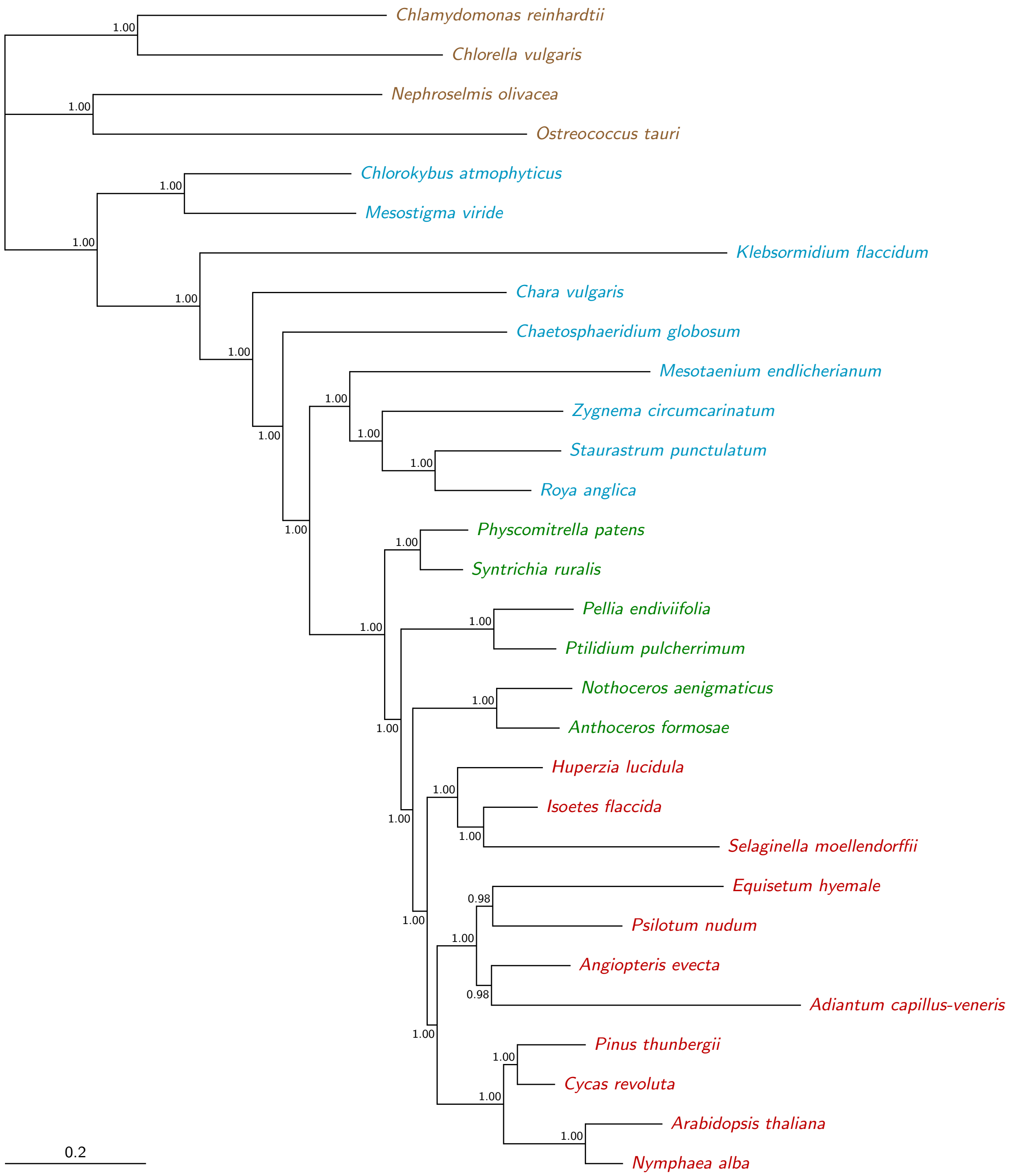

Figure S5 **Protein-coding gene data P4 MCMC NDCH2 (run 1 and run2 combined): GTR+Γ_4+NDCH2** Marginal likelihood: $-\overline{L}_h$ = 742934.6108. Posterior predicitive simulations of X^2: original statistic = 9116.6857, sample distribution= 7588.9545 to 8839.3090, p = 0.0000. 400,000 generations, 4,000 samples, 2,000 discarded as burnin. Mean tree length = 9.0904

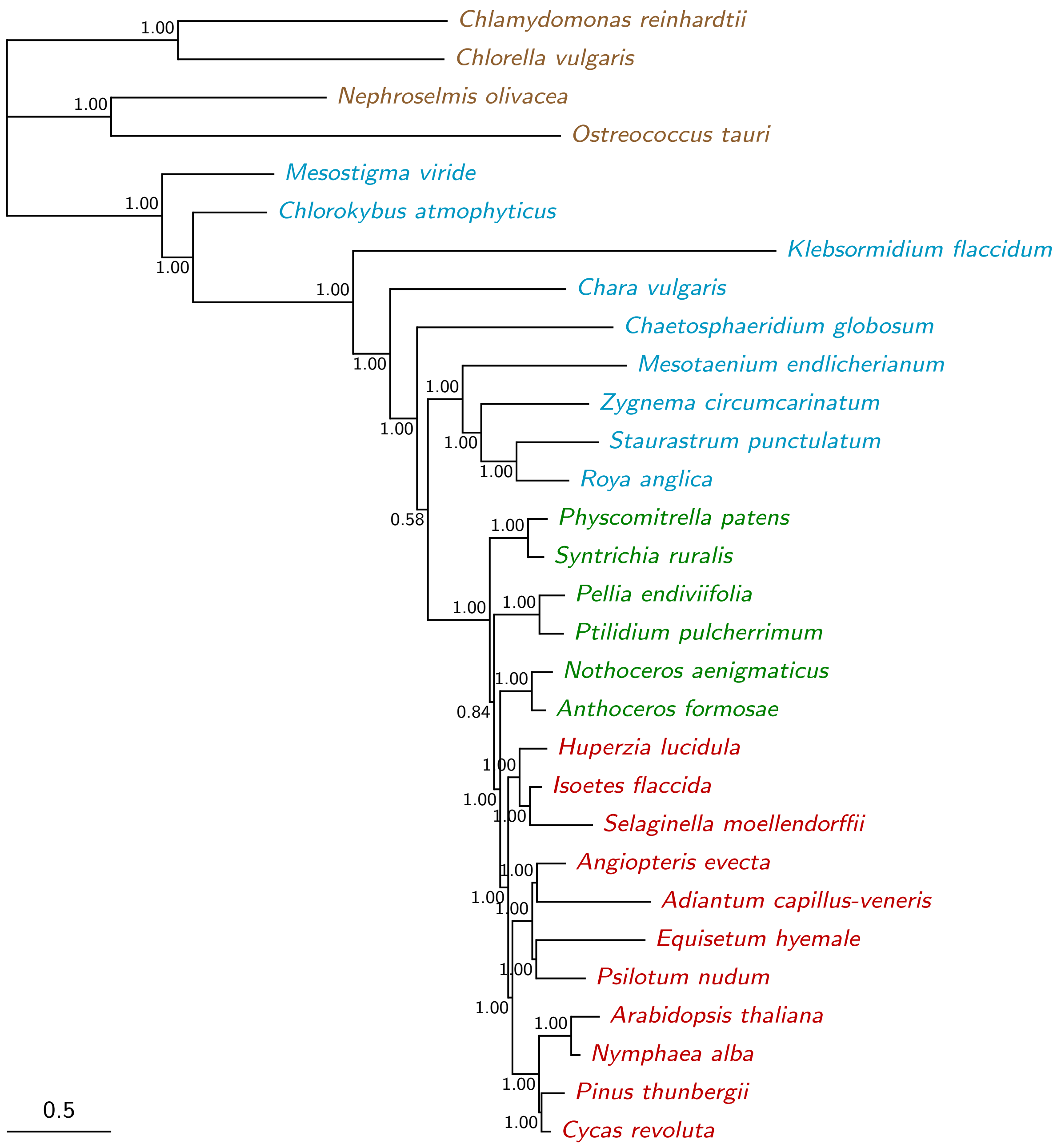

Figure S6 Protein-coding gene data Phylobayes MCMC: CAT-GTR+Γ_4 Run 1: Marginal likelihood: $-\overline{L}_h$ = 639966.6613, samples, 2,000 burnin. Run 2: Marginal likelihood: $-\overline{L}_h$ = 640027.9138, samples, 2,000 burnin Run 3: Marginal likelihood: $-\overline{L}_h$ = 640074.2916, samples, 2,000 burnin. Combined marginal likelihood: $-\overline{L}_h$ = 640034.343778, maxdiff = 0.104725, meandiff = 0.0024. Mean tree length = 20.5494

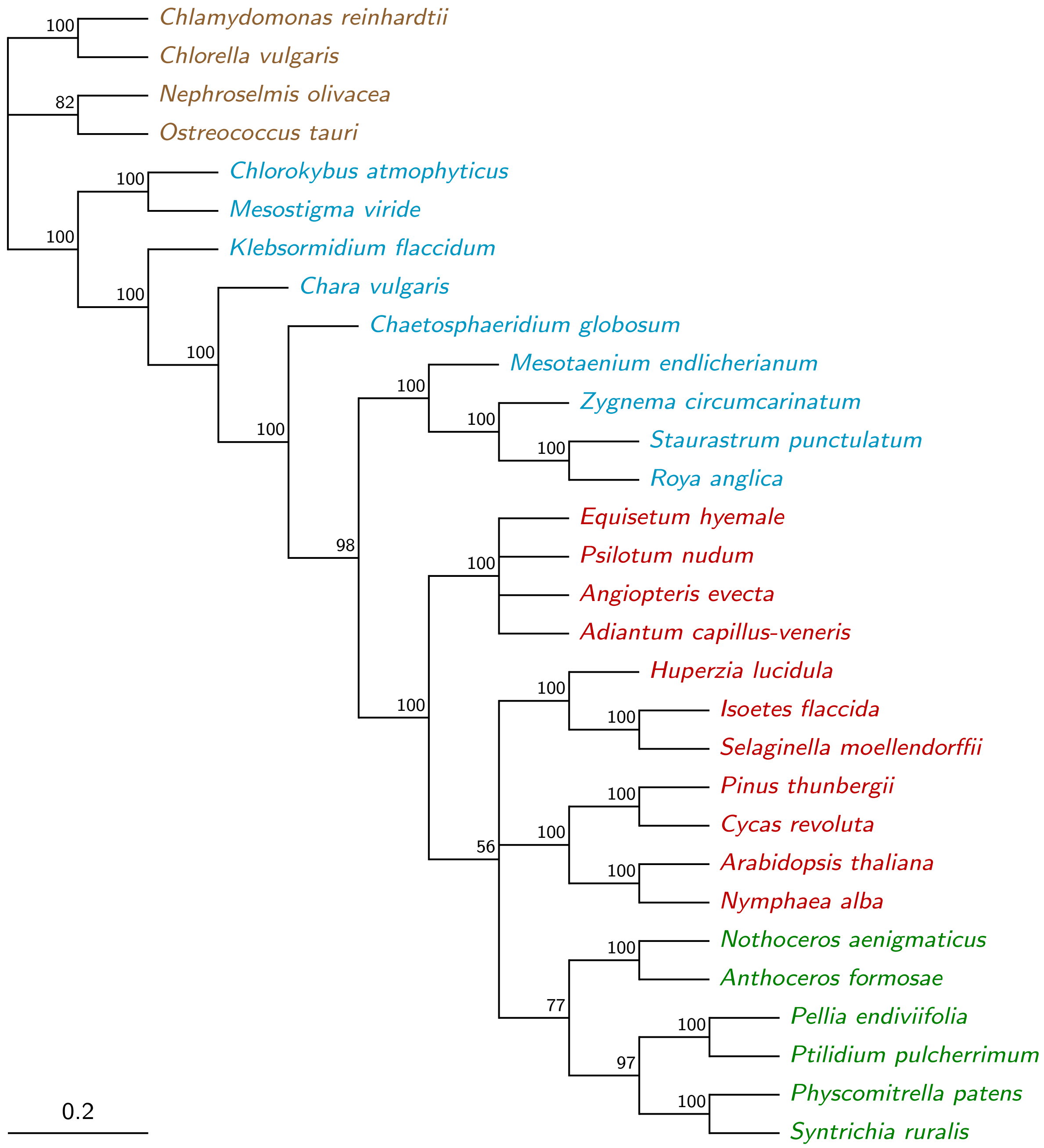

Figure S7 Protein data ML bootstrap: gcpREV+Γ+F_{mod} Optimal tree likelihood $-\overline{L}$ = 306636.8510. Tree length = 5.6983

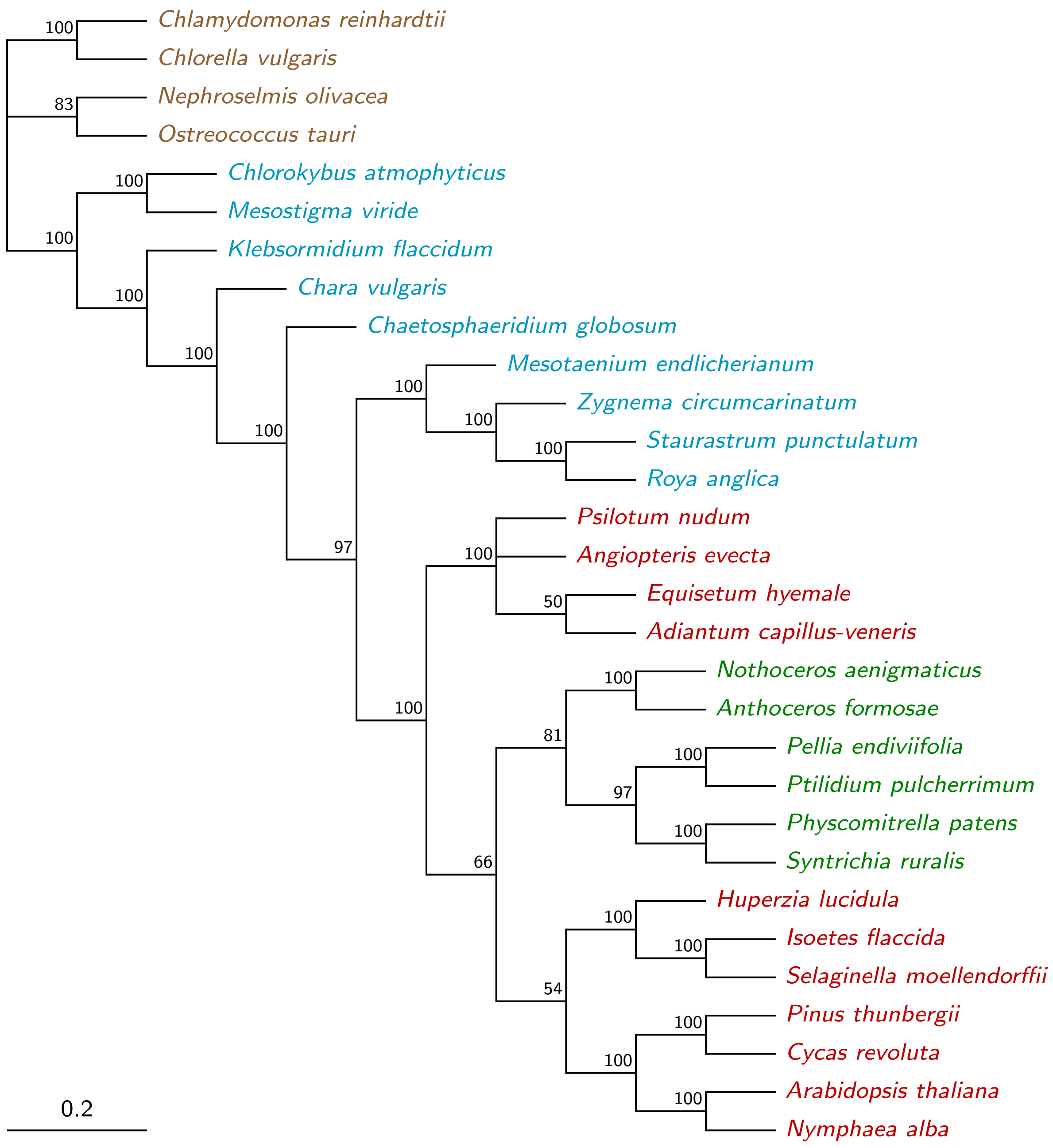

Figure S8 **Protein data ML bootstrap: cpREV+Γ_4+F_{est} and LG+Γ_4+F_{est}** 17 partitions as determined by PartitionFinder (all cpREV except where noted): (accd, ndha, ndhj, psbj, rpl2, rpl33, rps11, rps18) (atpa, ndhh) (atpb, psab, psbc, rps12) (atpe, atpf, ndhe, psai, psam, rps2, rps3, rps7, rps8, ycf12) (atph, chll, petb, psbf, psbt) (atpi, chlb, chln, ndhi, peta, psaj, psbz, rpl14, rpl16, rps19) (ccsa, ndhb, ndhg, rpl20, rpl21, rpl22, rpl23, rpl32, rps16) (cema, petl, rpoa, ycf4) (LG: clpp) (infa, petg, petn, psbh, psbi, psbm, psbn, rpl36, ycf3) (matk) (ndhc, ndhd, rpob, rpoc1, rpoc2, rps14, rps4) (ndhf, rps15) (ndhk, psaa, psac, psbd, psbe) (petd, psbb, psbk, psbl) (psba) (LG: rbcl); Optimal tree likelihood $-\overline{L}$ = 307856.9108. Length of optimal tree = 5.9500

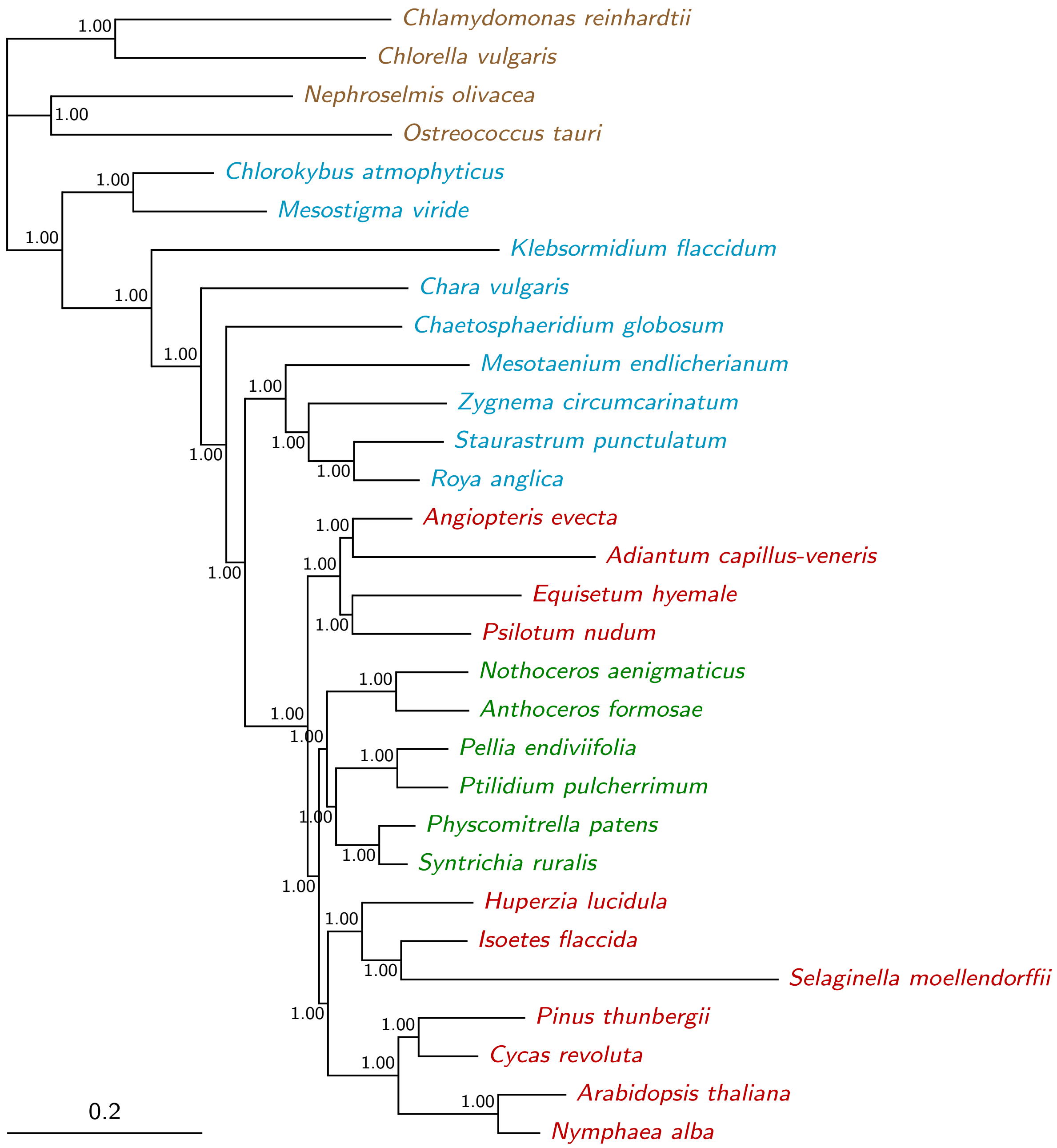

Figure S9 **Protein data P4 MCMC composition homogeneous: gcpREV+Γ_4+F_{est}** Marginal likelihood: $-\overline{L}_h$ = 306346.850333. Posterior predicitive simulations of X^2: original statistic = 3021.4465, sample distribution= 121.5581 to 216.3940, p = 0.0000. 2,000,000 generations, 20,000 samples, 10,000 discarded as burnin. Mean tree length = 5.5665

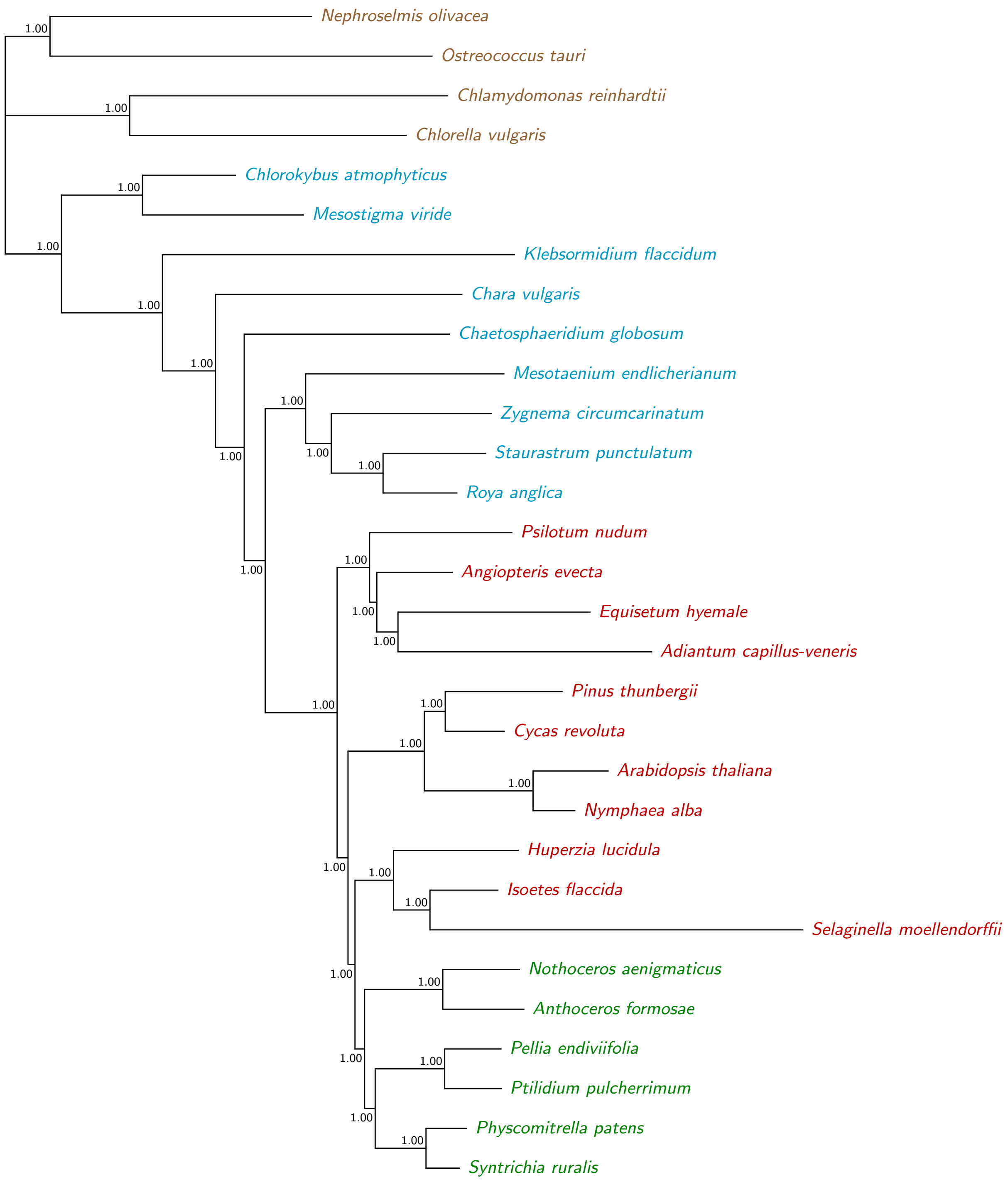

Figure S10 Protein data P4 MCMC NDCH2 Run3: gcpREV+Γ_4+F_{NDCH2} Marginal likelihood: $-\overline{L}_h$ = 302684.3298. Posterior predictive simulations of X^2: original statistic = 3021.4465, sample distribution = 2111.2480 to 3123.5135, p-value = 0.0002. 4,000,000 generations, 40,000 samples, 10,000 discarded as burnin. Mean tree length = 5.6475.

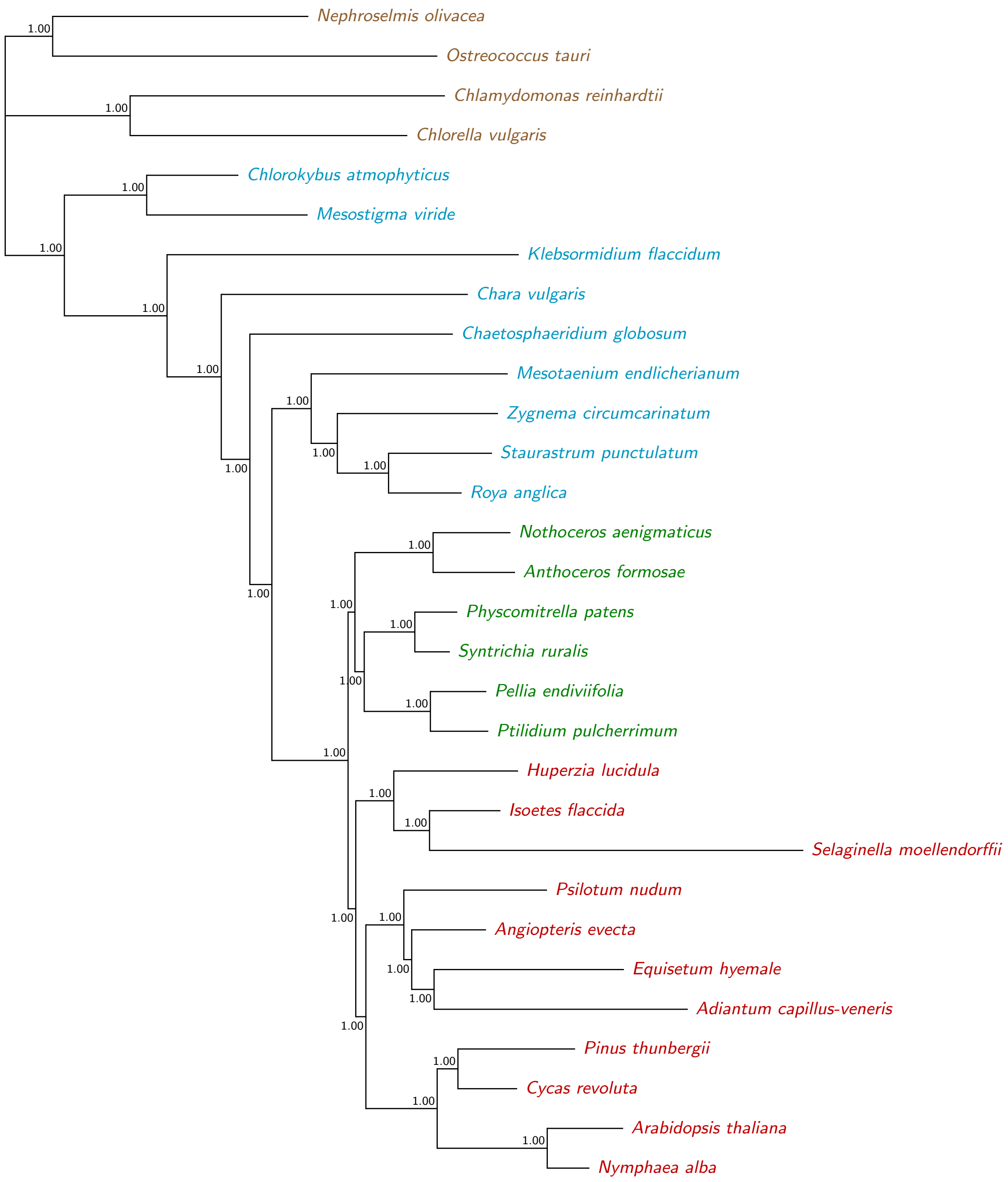

Figure S11 **Protein data P4 MCMC NDCH2 Run4: gcpREV+Γ_4+F_{NDCH2}** Marginal likelihood: $-\overline{L}_h$ = 302722.7301. Posterior predictive simulations of X^2: original statistic = 3021.4465, sample distribution = 2051.0655 to 3056.1848, p-value = 0.0026. 4,000,000 generations, 40,000 samples, 10,000 discarded as burnin. Mean tree length = 5.6575.

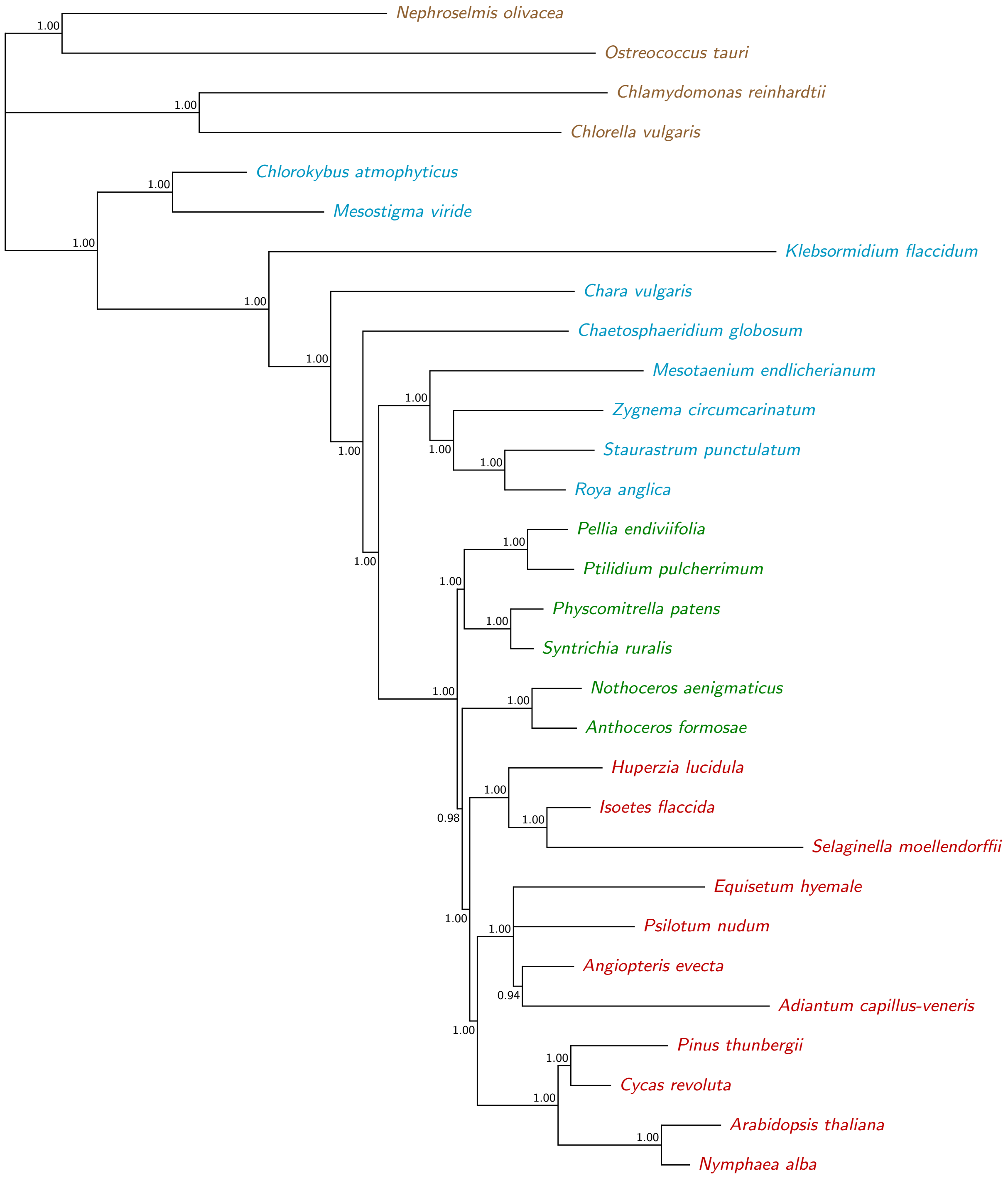

Figure S12 **Protein data Phylobayes MCMC: CAT+GTR+Γ_4** Run 1: Marginal likelihood: $-\overline{L}_h$ = 246839.9706, 26917 samples, 5,000 burnin. Mean tree length = 8.4076

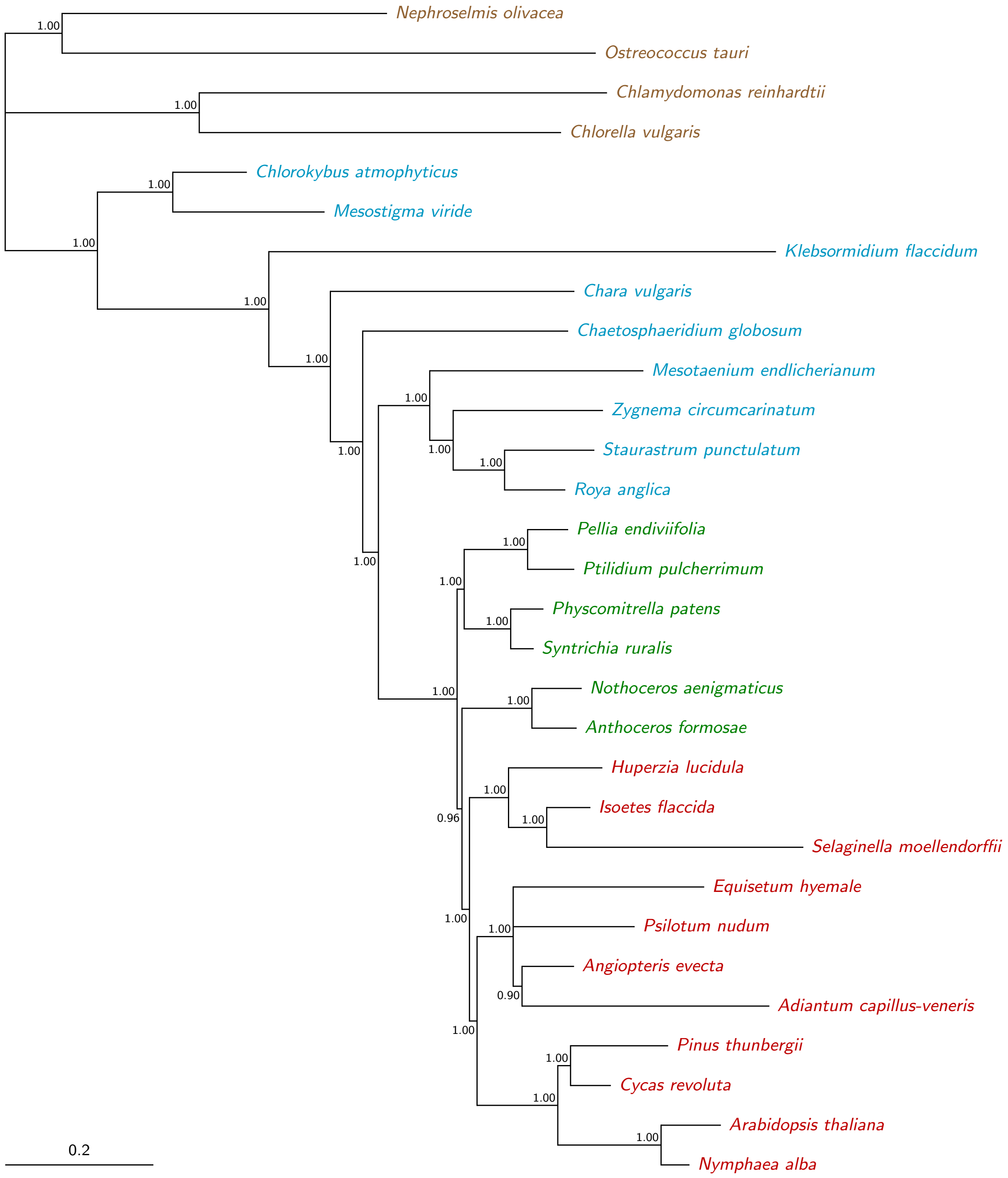

Figure S13 Protein data Phylobayes MCMC: CAT+GTR+Γ_4 Run 2: Marginal likelihood: $-\overline{L}_h$ = 247009.1536, 26876 samples, 5,000 burnin. Mean tree length = 8.4073

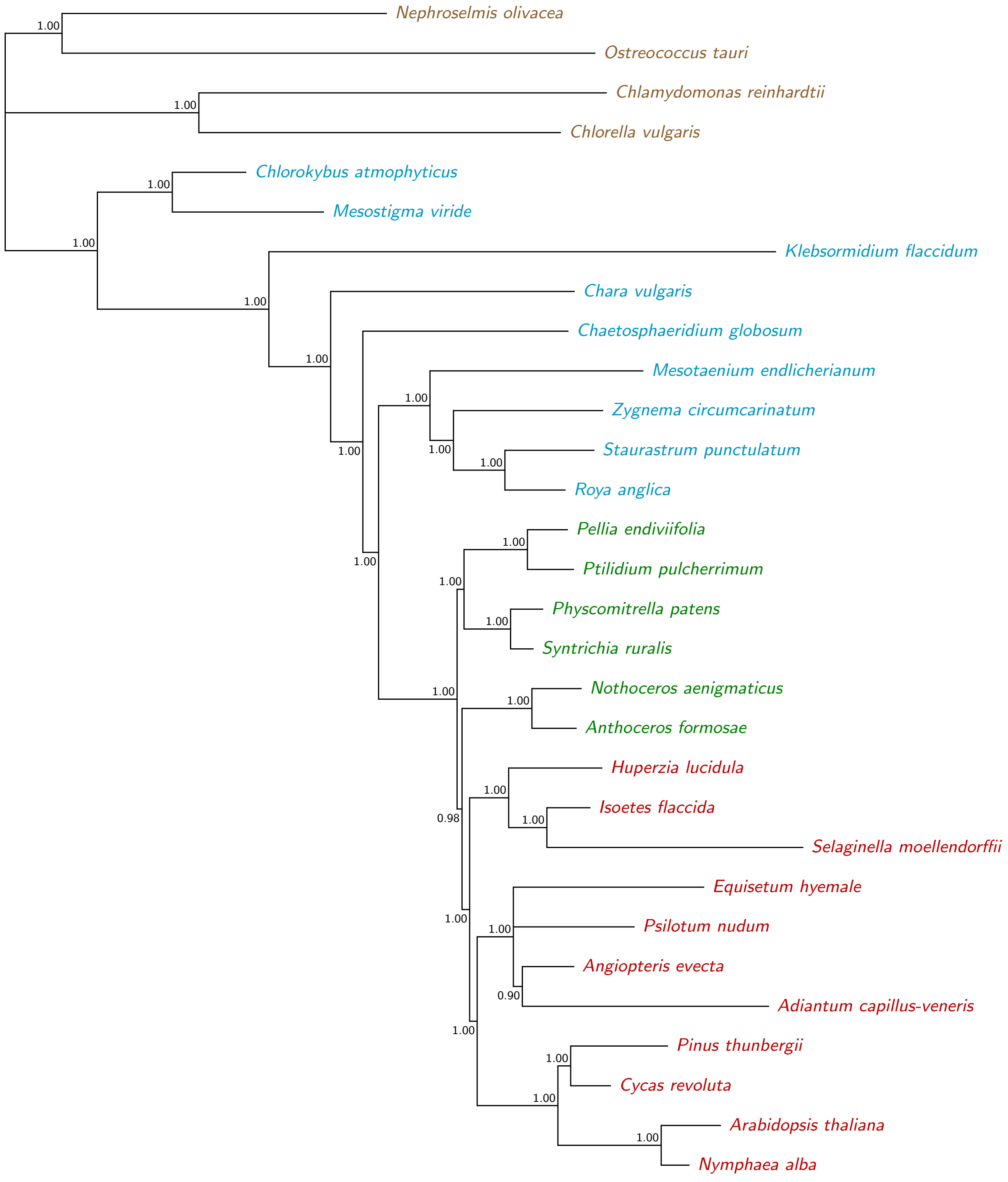

Figure S14 **Protein data Phylobayes MCMC: CAT+GTR+Γ_4** Run 3: Marginal likelihood: $-\overline{L}_h$ = 246797.2590, 26778 samples, 5,000 burnin. Mean tree length = 8.3978

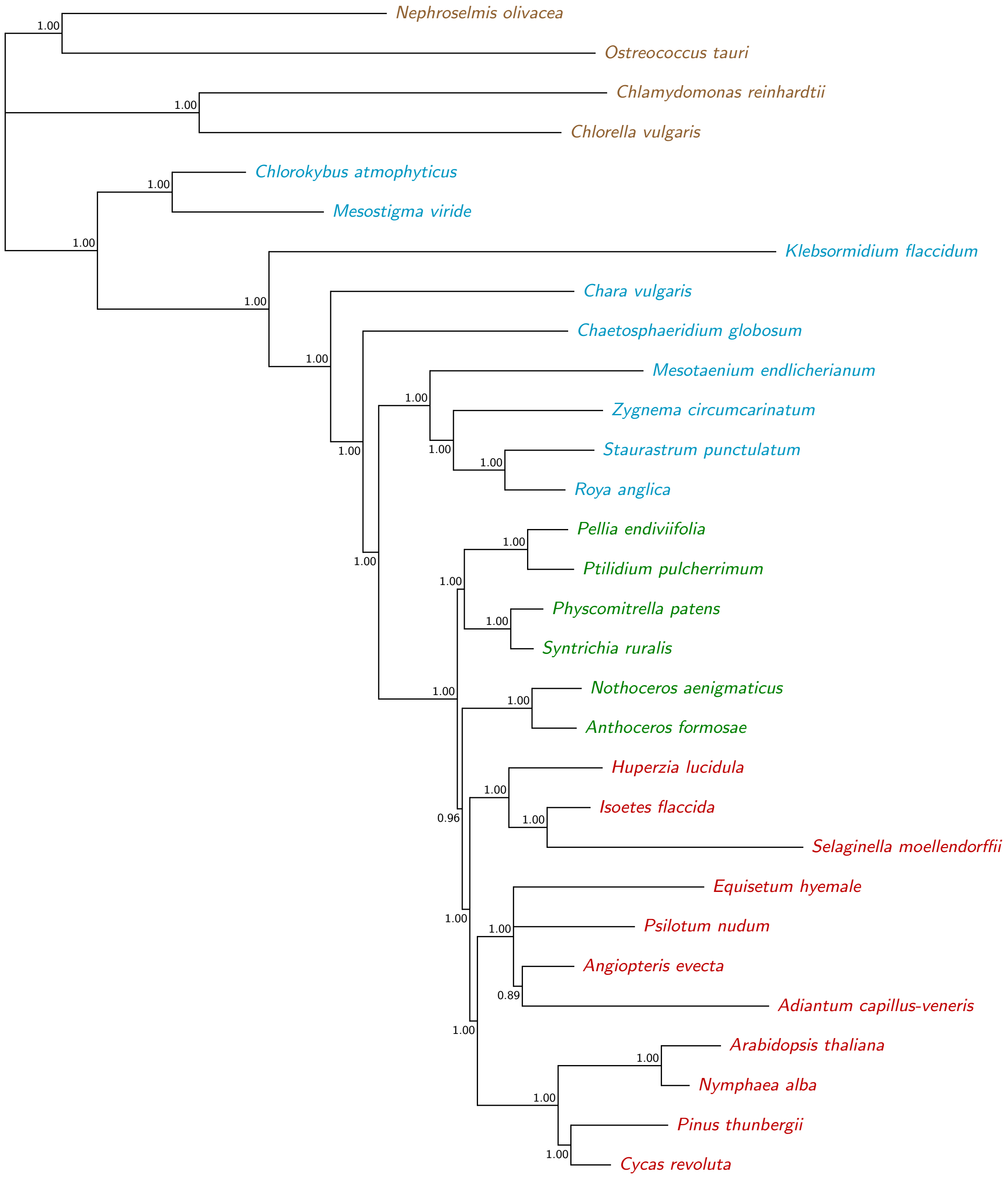

Figure S15 Protein data Phylobayes MCMC: CAT+GTR+Γ_4 Run 4: Marginal likelihood: $-\overline{L}_h$ = 246756.7830, 26771 samples, 5,000 burnin. Mean tree length = 8.4164
