# Supplementary material for: The Chloroplast Land Plant Phylogeny: Analyses Employing Better-Fitting Tree- and Site-Heterogeneous Composition Models
Source: Front Plant Sci. 2020 Jul 10;11:1062. doi: 10.3389/fpls.2020.01062 (PMC7373204; doi:10.3389/fpls.2020.01062)
Supplement: Supplementary file 2 [file DataSheet_2.pdf]

Table S1 Chloroplast gene sampling Gene nomenclature follows Stroebe et al. 1998 and online resource Chloroplast DB (http://chloroplast.cbio.psu.edu/tribedb.cgi).

Gene	nTax	nChar	πA	πC	πG	πT	%GC
accD	24	780	0.3093	0.1549	0.2183	0.3175	0.3731
atpA	30	1500	0.3120	0.1810	0.2131	0.2940	0.3941
atpB	30	1407	0.3028	0.1792	0.2220	0.2959	0.4012
atpE	30	369	0.3404	0.1707	0.2138	0.2751	0.3845
atpF	30	447	0.3726	0.1559	0.2022	0.2692	0.3581
atpH	30	243	0.2236	0.1960	0.2486	0.3318	0.4446
atpI	30	693	0.2704	0.1836	0.1892	0.3568	0.3728
ccsA	27	603	0.2548	0.1701	0.2031	0.3721	0.3731
cemA	28	726	0.3060	0.1610	0.1556	0.3774	0.3166
chlB	25	1437	0.3212	0.1647	0.2016	0.3125	0.3663
chlL	25	807	0.3048	0.1559	0.2219	0.3173	0.3778
chlN	25	1218	0.3107	0.1692	0.2121	0.3080	0.3813
clpP	30	546	0.3016	0.1634	0.2297	0.3052	0.3932
infA	24	213	0.3382	0.1503	0.2055	0.3059	0.3558
matK	20	393	0.3084	0.1835	0.1525	0.3556	0.3360
ndhA	25	909	0.2613	0.1704	0.2002	0.3681	0.3706
ndhB	26	1170	0.2664	0.1656	0.1817	0.3863	0.3473
ndhC	26	351	0.2488	0.1615	0.1962	0.3935	0.3577
ndhD	26	1377	0.2635	0.1727	0.1881	0.3757	0.3608
ndhE	26	279	0.2819	0.1543	0.1813	0.3826	0.3355
ndhF	25	1623	0.2528	0.1757	0.1973	0.3743	0.3730
ndhG	26	300	0.2297	0.1646	0.1847	0.4211	0.3493
ndhH	25	1134	0.3132	0.1532	0.2235	0.3102	0.3767
ndhI	26	426	0.3078	0.1583	0.1993	0.3347	0.3575
ndhJ	25	462	0.2877	0.1754	0.2130	0.3239	0.3884
ndhK	26	435	0.2817	0.1881	0.2071	0.3231	0.3952
petA	30	846	0.3269	0.1727	0.2031	0.2973	0.3758
petB	30	639	0.2513	0.1748	0.2096	0.3644	0.3844
petD	29	456	0.2656	0.1937	0.1958	0.3448	0.3895
petG	30	111	0.2428	0.1700	0.2127	0.3745	0.3827
petL	28	78	0.2555	0.1662	0.1424	0.4359	0.3086
petN	24	87	0.2045	0.1485	0.2663	0.3807	0.4148
psaA	30	2223	0.2560	0.1990	0.2143	0.3306	0.4133
psaB	30	2190	0.2531	0.1979	0.2093	0.3397	0.4072
psaC	30	243	0.2743	0.1765	0.2447	0.3044	0.4213
psaI	29	75	0.1849	0.2121	0.1397	0.4633	0.3518
psaJ	30	120	0.2400	0.2019	0.1744	0.3836	0.3764
psaM	21	84	0.2789	0.1412	0.1831	0.3968	0.3243
psbA	29	1056	0.2254	0.2296	0.2107	0.3342	0.4403
psbB	30	1524	0.2446	0.1788	0.2437	0.3329	0.4225
psbC	30	1419	0.2355	0.1873	0.2398	0.3374	0.4271
psbD	30	1059	0.2226	0.2010	0.2215	0.3549	0.4225
psbE	30	240	0.2615	0.2000	0.2093	0.3292	0.4093
psbF	29	102	0.2312	0.2292	0.1890	0.3506	0.4182
psbH	30	165	0.2496	0.1610	0.2238	0.3656	0.3848
psbI	30	108	0.2308	0.1863	0.1699	0.4130	0.3562
psbJ	30	84	0.2067	0.1810	0.2524	0.3599	0.4333
psbK	30	111	0.2114	0.2099	0.1829	0.3958	0.3928
psbL	29	114	0.2970	0.1975	0.1455	0.3600	0.3430
psbM	29	84	0.2652	0.2114	0.1071	0.4163	0.3186
psbN	30	120	0.2508	0.2192	0.1864	0.3436	0.4056
psbT	30	81	0.2144	0.1856	0.1815	0.4185	0.3671
psbZ	28	183	0.1975	0.1641	0.2028	0.4356	0.3669
rbcL	30	1425	0.2799	0.1925	0.2342	0.2934	0.4266
rpl14	29	366	0.3335	0.1599	0.2199	0.2866	0.3798
rpl16	29	387	0.3186	0.1914	0.2404	0.2496	0.4317
rpl2	29	780	0.3305	0.1835	0.2325	0.2535	0.4160
rpl20	29	303	0.3400	0.1789	0.1986	0.2825	0.3775
rpl21	19	279	0.3628	0.1474	0.2026	0.2872	0.3500
rpl22	25	303	0.3244	0.1862	0.1929	0.2965	0.3792
rpl23	28	213	0.3935	0.1581	0.1628	0.2855	0.3209
rpl32	24	129	0.3786	0.1828	0.1906	0.2481	0.3734
rpl33	25	168	0.4067	0.1530	0.1638	0.2764	0.3168
rpl36	30	102	0.3771	0.1529	0.2212	0.2487	0.3742
rpoA	28	537	0.3307	0.1729	0.1841	0.3123	0.3570
rpoB	28	2379	0.3222	0.1649	0.2127	0.3002	0.3776
rpoc1	29	1317	0.3090	0.1793	0.2042	0.3075	0.3835
rpoc2	29	1533	0.3212	0.1677	0.2106	0.3005	0.3782
rps11	30	351	0.3057	0.1900	0.2388	0.2654	0.4289
rps12	29	345	0.3283	0.1999	0.2167	0.2551	0.4166
rps14	30	288	0.3426	0.1783	0.2106	0.2685	0.3889
rps15	21	207	0.3665	0.1495	0.1682	0.3159	0.3177
rps16	16	231	0.3580	0.1584	0.1944	0.2892	0.3528
rps18	29	162	0.3670	0.1502	0.1661	0.3167	0.3163
rps19	30	249	0.3343	0.1801	0.1941	0.2916	0.3742
rps2	27	648	0.3416	0.1641	0.2036	0.2907	0.3676
rps3	29	441	0.3476	0.1637	0.2092	0.2794	0.3729
rps4	30	429	0.3336	0.1707	0.1904	0.3053	0.3612
rps7	30	456	0.3539	0.1794	0.2121	0.2547	0.3915
rps8	30	312	0.3468	0.1540	0.2116	0.2876	0.3656
ycf12	28	96	0.2626	0.1711	0.2068	0.3594	0.3780
ycf3	29	462	0.3512	0.1761	0.1934	0.2793	0.3696
ycf4	29	513	0.2776	0.1547	0.2210	0.3466	0.3758
